# The evolution of universal cooperation

**DOI:** 10.1126/sciadv.add8289

**Published:** 2023-02-17

**Authors:** Jörg Gross, Zsombor Z. Méder, Carsten K.W. De Dreu, Angelo Romano, Welmer E. Molenmaker, Laura C. Hoenig

**Affiliations:** ^1^ Department of Psychology, University of Zürich, Zurich, Switzerland.; ^2^ Institute of Psychology, Leiden University, Leiden, the Netherlands.; ^3^ Center for Research in Experimental Economics and Political Decision Making, University of Amsterdam, Amsterdam, the Netherlands.

## Abstract

Humans work together in groups to tackle shared problems and contribute to local club goods that benefit other group members. Whereas benefits from club goods remain group bound, groups are often nested in overarching collectives that face shared problems like pandemics or climate change. Such challenges require individuals to cooperate across group boundaries, raising the question how cooperation can transcend beyond confined groups. Here, we show how frequent intergroup interactions allow groups to transition from group-bound to universal cooperation. With frequent intergroup interactions, reciprocity of cooperative acts permeates group boundaries and enables the evolution of universal cooperation. As soon as intergroup interactions take place frequently, people start to selectively reward cooperation aimed at benefitting everyone, irrespective of their group membership. Simulations further show that it becomes more difficult to overcome group-bound cooperation when populations are fragmented into many small groups. Our findings reveal important prerequisites for the evolution of universal cooperation.

## Teaser

Opportunities to help outsiders reduce parochialism and facilitate the evolution of group-transcending, universal cooperation.

## INTRODUCTION

Human prosperity is deeply intertwined with group cooperation. In groups, humans create shared goods to the benefit of all its members, exceeding what individuals alone are capable of (*1*, *2*). Groups, however, rarely exist in isolation (*3*–*11*) and, in the course of history, people increasingly face global challenges like pandemics or climate change that require not only within-group cooperation but also cooperation that transcends group boundaries (*5*, *12*–*14*).

Already within groups, cooperation can be difficult to maintain (*14*–*16*). Cooperation is a costly action that benefits other group members, making cooperation exploitable. Group members who take advantage of the cooperation of others without cooperating themselves (i.e., free-riders) reap the benefits of cooperation without paying its costs. Furthermore, in repeated interactions, conditional cooperators (i.e., those willing to cooperate if others also do) often match others’ cooperation imperfectly. This can lead to a downward spiral of cooperation even in the absence of free-riders (*17*–*19*). Past research identified several mechanisms based on reciprocal interactions that provide bottom-up solutions to this social dilemma (*20*–*26*). Such reciprocity mechanisms are grounded in a simple and elegant idea: If cooperation provides benefits for a cooperator that outweigh its cost by, for example, being rewarded for cooperation by others, the payoff gap between cooperation and free-riding reverses, individual interests align with group interests, and cooperation in groups can emerge (*27*–*31*).

Whereas these mechanisms can explain how cooperation within groups is sustained, the unaddressed question is how cooperation can transcend group boundaries and establish public goods that benefit larger collectives. From the outset, group boundaries can skew the meeting probability of individuals, such that individuals are more likely to meet others belonging to their own group rather than other groups (*32*). Accordingly, solutions to social dilemmas based on reciprocal interactions may promote group-bound, parochial cooperation, but hinder cross-group cooperation (*32*–*37*).

However, group boundaries can also be fluid to different degrees (*5*, *38*–*40*). Exchanges across groups, as seen with increased globalization or in societies with greater relational mobility, increase the likelihood of intergroup encounters (*41*–*43*). Here, we provide a general framework for the evolution of universal cooperation and show that exchanges across group boundaries play a critical role for groups to transition from group-bound to universal cooperation. Experimentally, we show that cooperation is more likely to remain group-bound when group boundaries limit the likelihood to meet members of different groups. However, as soon as group boundaries become more fluid, allowing members to frequently interact with out-group members, reciprocity permeates group boundaries, and people start to enforce universal rather than parochial cooperation.

## RESULTS

**Fig. 1.**
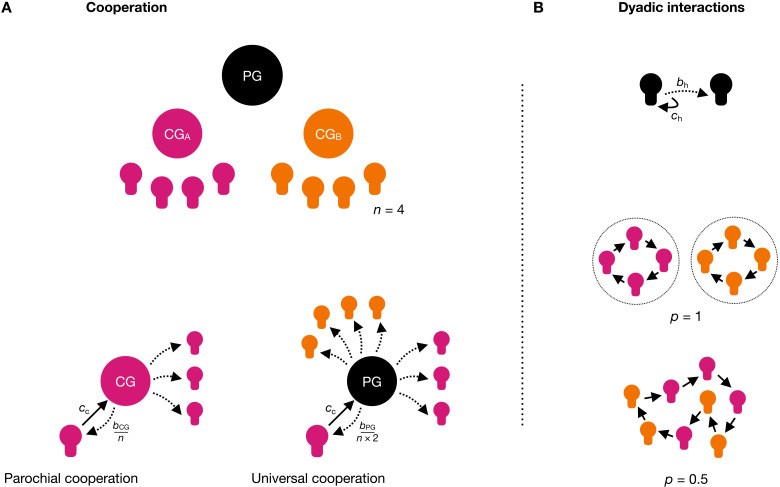
Modeling cooperation across group boundaries. Agents belong to different groups with their own club good (CG) and a group-independent, nonexcludable public good (PG) (**A**). Costly contributions to the club good (“parochial cooperation”) create a benefit *b*_CG_ for the group that the agent belongs to. Costly contributions to the public good (“universal cooperation”) create a benefit *b*_PG_ for all agents regardless of their group membership. In a second stage, agents interact in a dyadic exchange (**B**). In the role of the donor, an agent can pay a cost *c*_h_ to create a benefit *b*_h_ for another agent (their assigned receiver). Depending on the fluidity of group boundaries, agents may meet other agents for dyadic interactions only from their own group (*p* = 1) or may meet in- and out-group members with equal likelihood (*p* = 0.5).

For our theoretical analyses, we start with the simplest case of two groups consisting of *n* agents each. Both groups have their own local club good that exclusively benefits their group members. Next to the club goods, there is a public good that benefits all agents regardless of their group membership (Fig. 1A). Each agent can decide to contribute, at a cost *c*_c_, to their club good (i.e., to cooperate parochially/locally), to the public good (i.e., to cooperate universally), or to do neither (i.e., to free-ride). An agent’s contribution to the club good creates a benefit *b*_CG_ equally shared across every member of their respective group with *b*_CG_/*n* < *c*_c_ < *b*_CG_. Likewise, contributions to the public good create a benefit *b*_PG_ shared across all agents in the population with *b*_PG_/2*n* < *c*_c_ < *b*_PG_. This creates the conditions for a cooperation dilemma in both cases: Cooperation increases the overall welfare of the group or population, respectively (*c* < *b*), while the individual return for a cooperator is lower than the incurred cost (*b*_CG_/*n* < *c* and *b*_PC_/2*n* < *c*). If others do not cooperate, the best response is to not cooperate either (which is true for the club good and for the public good). If others cooperate, the best response is still not to cooperate because free-riding has a higher return for the agent than cooperation.

Hence, agents need an additional mechanism to outweigh the cost of cooperation. We consider a simple reciprocity mechanism that enables agents to reward each other for their actions. After the contribution stage, agents in the role of a “donor” meet another agent in the role of the “receiver” for a dyadic interaction in a simple helping game (Fig. 1B). The donor can pay a cost *c*_h_ to create a benefit *b*_h_ for the receiver. We assume that cooperative types reward the cooperation of others by extending help, while selfish types that attempt to free-ride on others in the contribution stage also do not help in these dyadic interactions because it would be costly for them to do so.

Previous theoretical models have shown that such a reciprocity mechanism can enforce group cooperation (*27*, *44*–*47*). Experiments further showed that helping is used to reward cooperators, which motivates free-riders to switch to cooperation (*28*, *29*, *48*, *49*). However, this reciprocity mechanism requires that agents regularly meet each other and can exchange conditional help. Belonging to a group can restrict who one meets and interacts with in dyadic exchanges. Depending on the fluidity of group boundaries [e.g., relational mobility (*38*)], agents may only meet in-group members or also have regular interactions with members from other groups.

We therefore model the likelihood to meet in-group versus out-group members with the parameter *p* (Fig. 1B). With *p* = 1, group boundaries are solid and dyadic interactions only occur within the boundaries of the group. With *p* = 0.5, group boundaries are fluid and meeting an in-group member is just as likely as meeting an out-group member. Our central question is whether and to which degree this meeting likelihood shifts the provision of group-exclusive club goods (parochial cooperation) to the provision of a truly encompassing public good (universal cooperation). For the simple case of two groups (with *c*_c_ = *c*_h_ = 1; *n* = 4; *b*_CG_ = 2; *b*_PG_ = 3), we performed exact numerical evolutionary simulations to calculate the long-term steady-state distributions based on the expected payoff of three strategy types: universal cooperators (who also help other universal cooperators), parochial cooperators (who help other parochial cooperators belonging to their group), and free-riders (who do not help anyone; see Materials and Methods for details and the Supplementary Materials for extensions of the parameter and strategy space).

**Fig. 2.**
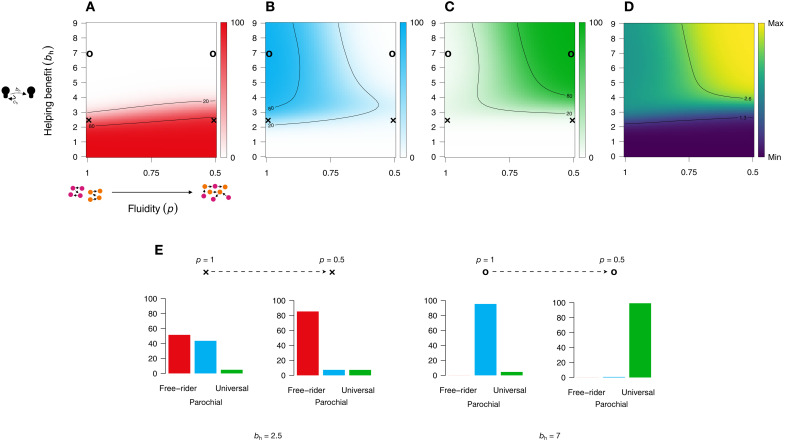
Evolution of universal cooperation. Proportion of free-riders (**A**), parochialists (**B**), universalists (**C**), and population welfare (i.e., agent earnings) (**D**) as a function of the benefit of helping (*b*_h_) and the likelihood to meet and interact with out-group members (fluidity *p*) based on numerical evolutionary simulations (*n* = 4; *c*_c_ = *c*_h_ = 1; *b*_CG_ = 2; *b*_PG_ = 3). (**E**) With low returns on helping, parochialism can emerge as long as group boundaries are solid [*b*_h_ = 2.5 and *p* = 1; left x marks in (A) to (C) show the position of this parameter combination in the full parameter space]. However, free-riders dominate when group boundaries are fluid [*b*_h_ = 2.5 and *p* = 0.5; right x marks in (A) to (C) show the position of this parameter combination in the full parameter space]. With large returns on helping, parochialism emerges under solid group boundaries [*b*_h_ = 7 and *p* = 1; left o marks in (A) to (C)], while universalism emerges when intergroup interactions frequently take place [*b*_h_ = 7 and *p* = 0.5; right o marks in (A) to (C)].

Figure 2 shows the evolution of universal cooperation. When the ability to reward cooperation through helping is low (low helping benefit *b*_h_), reciprocity cannot maintain any cooperation, and free-riding dominates (Fig. 2A). Cooperation begins to emerge when *b*_h_ exceeds 2. Specifically, with strict group boundaries (no group border fluidity; *p* = 1), cooperative types tend to proliferate as long as the share of either type of cooperator in the group exceeds the costs–to–net–benefits ratio of being a cooperator, *c*_c_/(*b*_h_ − *c*_h_). For completely fluid groups, cooperation can spread as long as either the share of universalists in the entire population or the share of parochialists within the group surpasses the same costs–to–net–benefits ratio.

If free-riders are driven out, the type of cooperation that dominates depends critically on the fluidity of group boundaries. When group boundaries do not allow cross-group interactions, parochial cooperators dominate (Fig. 2B). Even if one group is composed solely of universal cooperators, parochialists outcompete universal cooperation as long as their share in the other group is sufficiently large, i.e., when $\frac{n_{p}}{n}>\frac{b_{h}-c_{h}}{b_{h}-c_{h}+b_{\mathrm{CG}}}$. For example, with *b*_h_ = 3, *c*_h_ *=* 1, and *b*_CG_ *=* 2, if parochialists make up 50% of the opposing group, they benefit from their club good, take advantage of universal cooperation, and proliferate.

At the same time, populations in which universal cooperation can be sustained create more social welfare (since *b*_CG_ < *b*_PG_; Fig. 2D). Such populations emerge only when cross-group interactions are sufficiently likely (Fig. 2C). With completely fluid group boundaries, universal cooperators outcompete their fellow parochialists as long as the total number of universal cooperators is higher than the number of parochialists within a group. In particular, if universal cooperators reach a simple majority in the population (*n*_u_ > 50%), universal rather than parochial cooperation emerges (see the Supplementary Materials for detailed calculations). Also note that universal cooperation requires a higher *b*_h_ threshold than parochial cooperation; otherwise, free-riders dominate groups with completely fluid group boundaries (Fig. 2E). Under low helping benefit, solid group boundaries at least allow to sustain parochial cooperation, possibly explaining when and why groups would be motivated to restrict relational mobility.

To perform exact numerical simulations, we needed to focus on a set of simple strategy types (helping parochialists, helping universalists, and free-riders; see Materials and Methods). However, many other strategies are possible. For example, an agent may help other cooperators, regardless of their type of cooperation (“nondiscriminating helpers”), or agents may be willing to cooperate in stage 1, but not willing to help in stage 2, introducing a second-order free-rider problem. Previous research has highlighted the importance of considering extensive strategy spaces (*50*, *51*). We therefore ran additional agent-based simulations to investigate a broader strategy space that incorporates strategies like nondiscriminatory helping or second-order free-riding. These agent-based simulations revealed that discriminatory helping (i.e., helping only parochialists or universalists) is favored over nondiscriminatory helping and that second-order free-riding can crowd out cooperation if nonhelping cannot be detected and punished (i.e., by excluding second-order free-riders from receiving help). The latter is the case because nonhelping cooperators have a payoff advantage over helping cooperators, and their presence makes populations vulnerable to invasions by defectors. These additional analyses provide important boundary conditions of the reciprocity mechanism. The general pattern observed in our numerical simulations (Fig. 2) is recovered, as long as helping cooperators are able to detect nonhelping cooperators and abstain from helping them (see the “Conditional helping and second-order free-riding” section in the Supplementary Materials for details on these additional simulations).

Our theory generates simple and testable predictions: With higher likelihood to meet out-group members in dyadic exchanges (i.e., with higher fluidity), more public versus club good provision emerges, because more universal cooperation rather than parochial cooperation is rewarded and encouraged through dyadic interactions. Conversely, solid group boundaries favor the emergence of group-confined club good cooperation, and public goods are supported to a lesser degree, although they would be collectively more beneficial.

We tested these predictions in a preregistered interactive experiment. Two groups with four participants each were confronted with the social dilemma of group cooperation, universal cooperation, and free-riding. The experiment mimicked the general setup of the numerical simulations. Specifically, each participant had one unit in the first stage of each round that they could contribute to their group’s club good for a cost *c*_c_ = 1. This option created a benefit *b*_CG_ = 2 only for the group (marginal per capita return of 0.5). Alternatively, a participant could contribute their unit to the public good for the same cost creating a benefit of *b*_PG_ = 3 for all participants regardless of their group membership (marginal per capita return of 0.375). Each round concluded with a second stage in which participants were paired to one other participant for the helping game. In this second stage, each participant took the role of the donor and could help a receiver by paying another unit (*c*_h_ = 1) that created a benefit of *b*_h_ = 3 for the receiver. In the strict-boundary treatment, participants only interacted with other participants from their own group (*p* = 1) in this stage. In the fluid-boundary treatment, participants were randomly paired to other participants, regardless of their group affiliation (*p* = 0.5). Groups interacted repeatedly for 20 rounds, starting each round with their cooperation decision, followed by the dyadic helping stage (Fig. 1).

**Fig. 3.**
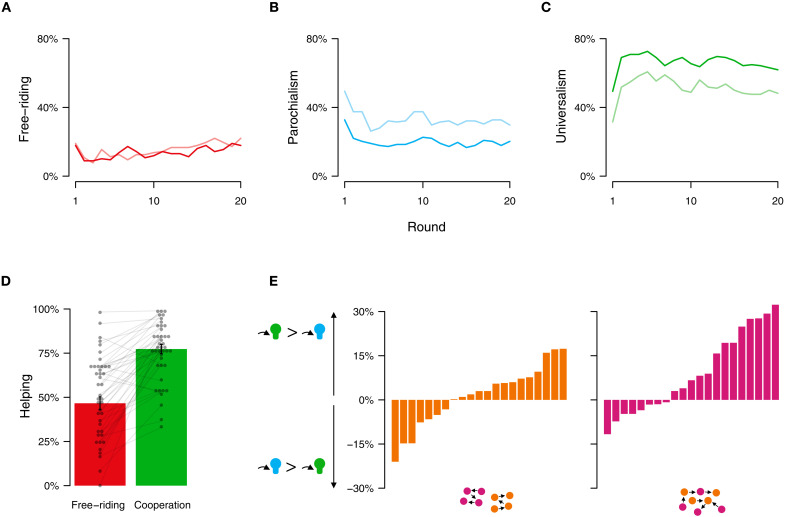
Emergence of universal cooperation in the laboratory. Free-riding was unaffected by border fluidity (red lines) (**A**). When dyadic interactions only took place within the boundary of the group (strict-boundary treatment, *p* = 1), participants exhibited more parochial, group-confined cooperation (light blue line) compared to when intergroup exchanges occurred frequently [fluid-boundary treatment, *p* = 0.5; (**B**) dark blue line]. Conversely, universal cooperation was more frequent in the fluid-boundary treatment (dark green line) compared to the strict-boundary treatment (**C**) (light green line). In general, participants extended more help toward cooperators than free-riders (**D**). However, group border fluidity influenced which type of cooperation was more likely rewarded in the dyadic exchanges. In the strict-boundary treatment, the average likelihood that a parochial cooperator received help was similar to that of a universal cooperator (**E**) (left). In contrast, the likelihood for a universal cooperator to receive help was larger than that of a parochial cooperator in the fluid-boundary treatment (E) (right). Each bar shows the percentage difference of help extended to universalists minus the help extended to parochialists in one of our groups. Negative numbers indicate a tendency toward rewarding parochial cooperation, whereas positive numbers indicate a tendency toward rewarding universal cooperation.

In line with our theoretical predictions, fluid group boundaries increased universal cooperation (multilevel logistic regression, *b* = 0.91, *p* = 0.02; Fig. 3C). The odds of universal cooperation increased by 148% when interactions across group boundaries took place. According to the fitted model, groups in the solid group boundary had a probability of 52% to cooperate universally, which increased to 73% in the fluid-boundary treatment. By contrast, solid group boundaries significantly increased the odds of parochial cooperation by 132% (multilevel logistic regression, *b* = 0.84, *p* = 0.004; Fig. 3B). According to the fitted model, groups in the solid group boundary had a probability of 37% to cooperate parochially, which decreased to 20% in the fluid-boundary treatment. The fluidity of group boundaries did not affect free-riding across treatments, in line with our numerical simulations (Fig. 3A). At the same time, we observed that universal cooperation was more frequent under solid group boundaries compared to our simulation results.

Helping decisions explained the observed shift from parochial to universal cooperation. Across both treatments, helping was conditional. Participants extended more help when the receiver was cooperating rather than free-riding (multilevel logistic regression, *b*_*p* = 0.5_ = 2.11, *p* < 0.001; *b*_*p* = 1_ = 1.61, *p* < 0.001; Fig. 3D). However, the type of cooperation that received more help shifted across treatments. In the solid-boundary treatment, participants were equally likely to help universal and parochial cooperators, on average (multilevel logistic regression, *b* = 0.09, *p* = 0.53; Fig. 3E, left). However, when interactions took place across group boundaries, universal cooperators were more likely to receive help compared to parochial cooperators (*b* = 0.81, *p* < 0.001; Fig. 3E, right). With frequent intergroup interactions, a norm of universal cooperation was more strongly enforced, resulting in more public good provision and reducing locally confined parochial cooperation. Helping decisions were sensitive not only to the type of cooperation that the receiver exhibited in stage 1, but also whether the receiver had helped before. This is in line with the assumption of the numerical and additional agent-based simulations that helpers condition their helping choice on a combination of stage 1 and stage 2 behavior of the receiver and punish second-order free-riding by omitting help (see tables S3 and S4 in the Supplementary Materials).

**Fig. 4.**
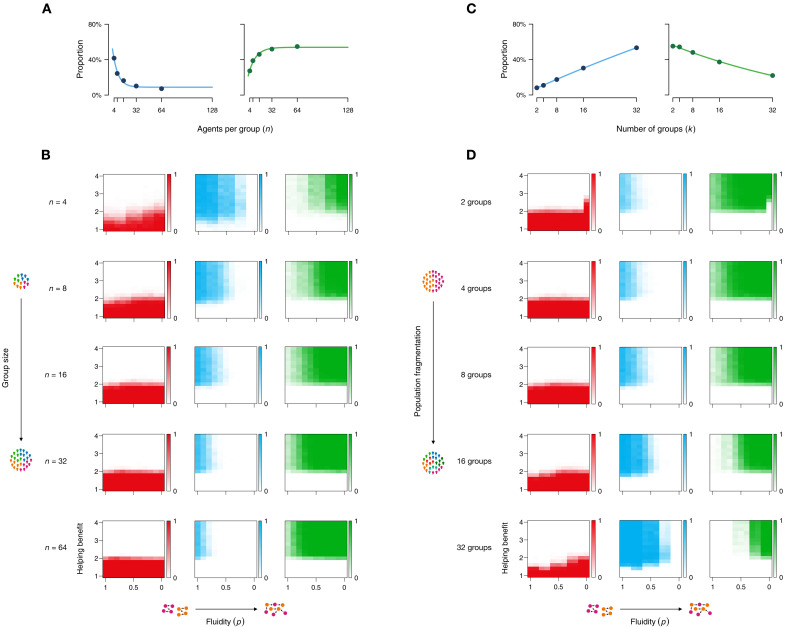
Group size and population fragmentation. With larger groups, parochial cooperation decreases (**A**) (left), and universal cooperation increases (A) (right); based on *k* = 8 groups with *n* = [4, 8, 16, 32, 64] agents per group averaged across (A) or separated by (**B**) *p* = [1.0, …, 0.0] and *b*_h_ = [1, …, 4] with *c*_c_ = *c*_h_ = 1, *b*_CG_ = 2, *b*_PG_ = 3, and μ = 10^−4^. When populations are segregated into more groups, parochial cooperation is favored (**C**) (left), and universal cooperation is less frequently established (C) (right); based on *N* = 128 split into *k* = [2, 4, 8, 16, 32] groups, averaged across *p* = [1.0, …, 0.0] and *b*_h_ = [1, …, 4]. Across the whole parameter space (with *c*_c_ = *c*_h_ = 1, *b*_CG_ = 2, *b*_PG_ = 3, and μ = 10^−4^), it can be seen how a higher fragmentation of the population into more subgroups leads to more parochial cooperation when *p* > 0.5 (**D**). Parochial cooperation is also more resistant to free-riding, i.e., it emerges under lower levels of *b*_h_ with higher population fragmentation.

Results demonstrate how intergroup interactions allow for the evolution of universal cooperation. So far, however, our model and experiments only focused on the basic case of two (small) groups. Social groups, however, can harbor many individuals, and populations can consist of more than two groups. To extend our results to larger groups and more complex population structures, we performed additional agent-based simulations (see Materials and Methods for details). Our first result shows that larger groups actually favor the emergence of universal cooperation. As the size of groups increases, club goods disappear, and groups start to establish group-transcending public goods even under more solid group boundaries (Fig. 4, A and B). Conversely, for many small groups, it is more difficult to establish cross-boundary universal cooperation. To see this, we investigated populations that were less or more fragmented, meaning that they are segregated into fewer and large groups or many small groups. With higher fragmentation, parochial cooperation increases (Fig. 4C). With every additional group in the population, the percentage of universalists decreases by 1.2%. Hence, it is more difficult to establish public goods when a population consists of many small groups. More fragmented populations have, however, also an advantage in that they can establish club good cooperation already under low levels of *b*_h_ without getting invaded be free-riders (Fig. 4D).

## DISCUSSION

Here, we showed how universal cooperation can evolve. Our three complementary approaches—numerical evolutionary simulations, a behavioral experiment, and agent-based simulations—identify intergroup interactions as a key determinant for the emergence of public goods provision. While dyadic reciprocity can maintain cooperation toward both club and public goods, group boundaries determine what type of cooperation emerges. When reciprocal interactions remain group bound, so does cooperation. As soon as reciprocity can permeate group boundaries, groups become more likely to support shared public goods and neglect their own club goods.

This process can also provide a framework for understanding fission-fusion dynamics of groups (*52*). With the establishment of public goods, group characteristics (i.e., club goods) disappear, and groups merge to create larger collectives. Indeed, participants in our experiment reported that they identified more with the collective than their group when reciprocal interactions took place across group boundaries [fig. S4; see also (*53*, *54*)]. Through this process of interactions across group boundaries, individuals in small groups not only shift from parochial to universal cooperation, but also slowly come to perceive themselves as part of larger, overarching collectives with truly encompassing public goods to care for.

In the experiment, we also observed a large degree of universal cooperation, even under solid group boundaries. Additional exploratory analyses showed that decisions to opt for universal cooperation were related to individual-level social preferences (fig. S3). Regardless of the treatment, more prosocial participants had a higher likelihood to cooperate across group boundaries [see also (*3*)], while social preferences were unrelated to parochial cooperation. This suggests that social preferences may, to some degree, help to establish group-transcending cooperation, independent of the structural characteristics of groups (i.e., *p*).

Previous research has shown how individuals can fail to solve shared collective action problems when they have the ability to tackle such problems on their own, even if such “self-reliance” is less efficient (*1*, *55*, *56*). Only when individual members become more interdependent and, concomitantly, less able to solve shared problems on their own will group cooperation emerge. Here, we extend these findings to the intergroup context. With lower interdependence across groups (operationalized as a lack of intergroup interactions), group-bound cooperation is favored, while more efficient universal cooperation suffers. Similar to the transition from self-reliance to group cooperation (*1*, *55*, *56*), higher interdependence across groups (operationalized as more frequent interactions across group boundaries) promotes a transition from localized to more efficient global cooperation.

Especially with global challenges like climate change or pandemics that require cooperation across group boundaries, more fluid group boundaries and increased exchange across group borders can help to establish public goods from “bottom-up” without requiring a central authority or complex institutions to curtail free-riding. Globalization, characterized by increased trade relations and mobility of individuals (*5*, *43*) and the integration of nation-states into larger unions, may therefore help to create global public goods, supported by reciprocal relationships between members of different groups.

## MATERIALS AND METHODS

### General setup

#### Nested social dilemma

To investigate cooperation across group boundaries, we consider populations in which each agent belongs to one group. Groups have a shared club good, and only group members can provide for this club good and benefit from it. Hence, the club good is nonrivalrous (i.e., it benefits group members equally) and excludable (i.e., agents that belong to group *y* do not benefit from the club good from group *x*). Next to each group’s club good, there exists one public good that is not only nonrivalrous (i.e., it benefits everyone equally) but also nonexcludable [i.e., no one can be excluded from its benefits; see also (*4*, *7*, *9*–*11*, *53*)]. Each agent is endowed with a private resource or endowment *e* set to 1 and can invest this resource to their group’s club good, or to the public good, or keep it for private consumption. Allocating the endowment to the club good generates a benefit *b*_CG_ that is equally shared across all members of the group. Allocating the endowment to the public good generates a benefit *b*_PG_ that is equally shared across all agents regardless of their group affiliation. Both of these actions can be classified as cooperation, because we assume that (i) the individual returns are smaller than 1, which means that the individual return from providing own resources for the club good or public good is less than keeping the resource for private consumption, and (ii) *b*_CG_ and *b*_PG_ are larger than 1, and thus, cooperation creates a net benefit for others that exceeds the value of private consumption. This creates the social dilemma of cooperation: Free-riding is always more beneficial for the individual, while cooperation increases the total welfare of each group (in the case of the club goods) or global welfare (in the case of the public good).

#### Dyadic interactions

In the second dyadic interaction stage, every agent is assigned a partner and receives another endowment of one unit. They can decide to “help” their partner or not. In the former case, they lose their unit but create a benefit *b*_h_ for their partner. Whom agents meet in these dyadic interactions is governed by *p*, the probability to be paired with an in-group member. With *p* = 1, group members only meet other members from their own group (i.e., group boundaries are solid, and group members do not meet out-group members). With *p* = 0.5, the likelihood to interact with an in-group member is as likely as the likelihood to interact with an out-group member. Hence, *p* governs the probability of cross-group interactions or, in other words, the fluidity of group boundaries.

### Numerical simulations

#### Expected payoffs of different strategies

To analyze the emergence of parochial (i.e., club good) cooperation and universal (i.e., public good) cooperation, we performed evolutionary numerical simulations to derive the long-term steady-state distribution of different strategies. For these simulations, we defined three strategy types: universal cooperators (*U*) that invest their resource into the public good and help other agents that cooperate universally when meeting them in stage 2, parochial cooperators (*P*) that invest their resource into their group’s club good and help other parochial agents of their own group, and free-riders (*F*) that do not cooperate and do not help.

Assume that there are *k* = 2 groups of size *n* each, the total number of agents being *N* = *kn*. We denote the number of agents in group *i* as *n_i_*. Universal or parochial cooperation creates a cost *c*_c_ for the cooperating agent. Parochial (or club good) cooperation creates a benefit *b*_CG_ with *b*_CG_/*n_i_* < *c*_c_ < *b*_CG_. Universal (or public good) cooperation creates a benefit *b*_PG_ with *b*_PG_/*N* < *c*_c_ < *b*_PG_. The parameter *p* denotes the likelihood to meet an in-group member in stage 2 (with 1 − *p* denoting the likelihood to meet an out-group member). Helping creates a benefit *b*_h_ for the receiver and a cost *c*_h_ for the helping agent.

This leads to the following expected payoff functions of these three types, respectively

$$\begin{array}{rl} \pi_{Ui} & =2e+\overset{\text{PG return}}{\overbrace{\frac{n_{U}\times b_{\mathrm{PG}}}{N}}}+\overset{\text{CG return}}{\overbrace{\frac{n_{Pi}\times b_{\mathrm{CG}}}{n_{i}}}}\;\underset{\;\text{Cost of cooperation}}{\underbrace{-c_{c}}}\\ & \;+\overset{\text{Likelihood to meet another}\;U}{\overbrace{\left(p\times \frac{n_{Ui}-1}{n_{i}-1}+(1-p)\times \frac{n_{U}-n_{Ui}}{N-n_{i}}\right)}}\\ & \;\times \;\underset{\mathrm{Cost}/\mathrm{benefit}\;\mathrm{of}\;\mathrm{helping}}{\underbrace{\left(b_{h}-c_{h}\right)}}\\ \pi_{Pi} & =2e+\frac{n_{U}\times b_{\mathrm{PG}}}{N}+\frac{n_{Pi}\times b_{\mathrm{CG}}}{n_{i}}-c_{c}\\ & \;+\;\overset{\text{Likelihood to meet another}\;P}{\overbrace{\left(p\times \frac{n_{Pi}-1}{n_{i}-1}\right)}}\;\times (b_{h}-c_{h})\\ \pi_{Fi} & =2e+\frac{n_{U}\times b_{\mathrm{PG}}}{N}+\frac{n_{Pi}\times b_{\mathrm{CG}}}{n_{i}} \end{array}$$

Note that the returns from the public good and club good are the same for all agents in group *i*, except that cooperating agents pay a cost *c*_c_ on which the selfish agent is free-riding. It is therefore easy to see that cooperation in stage 1 is dominated by free-riding. Stage 2 therefore can be considered as our cooperation-enforcing mechanism as long as the expected net benefit from dyadic helping is larger than the incurred cost of cooperation.

#### Evolutionary dynamics

We study the success and transmission of strategies through an evolutionary process. After each generation (i.e., after the conclusion of stage 2), one agent is randomly selected to either adopt a new strategy type randomly (random mutation; with probability μ) or to copy a type of the population depending on their success or fitness *e*^π^ (selection; with probability 1 − μ). Hence, after each iteration, a single agent changes its type to one from the type space or adopts the strategy of another agent in the population, depending on their strategy’s success. This frequency-dependent Moran process can be interpreted as modeling natural selection or social learning. The underlying idea is that strategies with higher payoffs are more likely to be adopted by other agents and spread in the population, while strategies with relatively low payoff die out in the long run. Furthermore, new strategies can emerge randomly (with μ), which can be interpreted as mutation (in the case of genetic evolution) or experimentation (in the case of social learning).

The probability that an agent in group *i* of type *j* is selected to adapt their strategy (the “dying” or adapting agent) is given by

$$p(x_{ij}\to )=\frac{1}{k}\times \frac{n_{ij}}{n}$$

The adoption process occurs globally, i.e., agents that are selected for adoption switch to a strategy chosen from the entire population based on their current success. The probability that the adopting agent switches to type *y* is

$$p(\to y)=\frac{\sum_{s=1}^{k}n_{sy}e^{\pi_{sy}}}{\sum_{t=1}^{l}\sum_{s=1}^{k}n_{st}e^{\pi_{st}}}$$

Thus, the probability that an agent in group *i* having type *j* switches to type *y* can be calculated as

$$p(x_{ij}\to y)=\frac{i}{k}\times \frac{n_{ij}}{n}\times \frac{\sum_{s=1}^{k}n_{sy}e^{\pi_{sy}}}{\sum_{t=1}^{l}\sum_{s=1}^{k}n_{st}e^{\pi_{st}}}$$

The state vector **w** specifies the frequency of types in each group

$$w=[w_{11}w_{12}\ldots w_{1l}\ldots w_{kl}]=\left[\frac{n_{11}}{N}\frac{n_{12}}{N}\ldots \frac{n_{1l}}{N}\ldots \frac{n_{kl}}{N}\right]$$

The transitions matrix **M** describes the transition probabilities between states. Specifically, when agent *x_ij_* switches to some other type *y* ≠ *j*, there is a transition between states

$$\left[\ldots \frac{n_{ij}}{N}\ldots \frac{n_{iy}}{N}\ldots \right]$$

and

$$\left[\ldots \frac{n_{ij}-1}{N}\ldots \frac{n_{iy}+1}{N}\ldots \right]$$

The probabilities of such a transition are given by *p*(*x_ij_* → *y*), which allows us to construct the complete transition matrix **M**.

The eigenvector with the largest eigenvalue of this transition matrix gives the long-term steady-state distribution of the population. Figure 2 is based on 4641 numerical simulations, calculating the steady-state distribution based on *k* = 2, *n* = 4, *c*_c_ = *e* = *c*_h_ = 1, *b*_CG_ = 2, *b*_PG_ = 3, and μ = 10^−4^ and for *p* = [0.5, 0.51, …, 1.0] and *b*_h_ = [0, 0.1, …, 9]. In the Supplementary Materials, we further report numerical simulation results on how the population composition is affected by changes in *b*_CG_ and *b*_PG_.

### Behavioral study

#### Sample and preregistration

In total, 336 participants took part in the experiment. The experiment took place at the Leiden Social Interaction laboratory in a large room in front of personal computers with cubicles that are separated by divider walls, such that people cannot see each other once seated. Because of a coronavirus disease lockdown, we additionally collected 11 groups on the online research platform Prolific. To do so, we adapted the experiment (which was already programmed in HTML/PHP and jQuery) by adding a chat functionality. This allowed participants to contact the experimenters during the experimental session and ask questions while reading the instructions or answering comprehension questions (similar to the laboratory environment; communication was only possible between participant and experimenter but not between participants). The study was approved by the ethics committee of the Department of Psychology of Leiden University (file 2021-05-26-J.A.J.Gross-V2-3250). Sample sizes, experimental setup, and hypotheses were preregistered (https://aspredicted.org/LK7_VH5 and https://aspredicted.org/VY8_C51). Following our theoretical results, we preregistered two main hypotheses: (i) Dyadic interactions across groups will increase universal cooperation compared to dyadic interactions that only take place between group members. (ii) Participants will more likely reward universal cooperation when interactions take place across groups (*p* = 0.5) compared to when interactions stay groupbound (*p* = 1). We set out to test 20 groups per treatment. We tested two more groups than preregistered due to a calculation mistake when we moved data collection to the online environment Prolific. However, removing the last two tested groups from the analyses does not change our results.

#### General implementation

Eight participants were randomly split into two groups of four participants. After reading and signing informed consent forms, participants received instructions for the main task. They were informed that they were randomly assigned to one of two groups and that they would make decisions that would affect their own payoff for this study as well as the payoff of the other participants of the two groups. After the instructions, extensive comprehension questions made sure that participants had sufficient understanding of the rules of the task.

The task consisted of two stages that were repeated for 20 consecutive rounds. In the first stage, each participant received 1 monetary unit (“MU”) worth €1 and had to simultaneously decide whether to invest this MU to the “public pool” (i.e., universal/public good cooperation), or to their group’s “group pool” (i.e., parochial/club good cooperation), or keep the MU. Each MU invested to the public pool was multiplied by 3 (*b*_PG_) and divided equally across all eight participants. Hence, if everybody decided to contribute their MU to the public pool, each participant would earn 8 × 3/8 = 3 MUs. However, if one participant instead decided to keep their MU (i.e., free-ride), this participant would earn 7 × 3/8 + 1 = 3.625 MUs, while the other group members only earned 7 × 3/8 = 2.625 MUs. Every MU invested to the group pool was multiplied by 2 (*b*_CG_) and divided equally across members of the group that the group pool belonged to. Again, free-riding would yield a higher return than parochial cooperation. For example, if all group members decided to contribute their MU to the group pool except for one participant, the three cooperating group members would earn 3 × 2/4 = 1.5 MUs, while the free-riding group member would earn 3 × 2/4 + 1 = 2.5 MUs.

Note that this nested social dilemma also creates exploitation opportunities across groups. For example, if all members of group *x* would invest their unit into the public pool, while all members of group *y* would invest their unit into their group pool, members of group *x* would only earn 4 × 3/8 = 1.5 MUs, while members of group *y* would earn 4 × 3/8 + 4 × 2/4 = 3.5 MUs.

After participants had made their stage 1 decisions, they received feedback on (i) how many MUs were contributed to the public pool and their group pool, (ii) their return from the public and group pool, (iii) their total earnings, and (iv) the average earnings of other participants from their group and the other group. Then, they moved to stage 2. In stage 2, they received another MU in the role of the “decider,” were assigned to another participant (the receiver), and had to decide whether to keep this MU for themselves or transfer it to the other participant. In the latter case, the receiver received 3 MUs (*b*_h_). Before making this decision, participants were informed about the group affiliation of their receiver and their stage 1 decision (i.e., whether this person decided to keep their MU or contribute it to their group pool or to the public pool). From round 2 onward, participants also learned whether their receiver decided to transfer their MU in stage 2 in the previous round, as well as their receiver’s cooperation decision in the last round (i.e., second-order reputation information). After every participant made their stage 2 decision, they received feedback on whether the participant that was in the role of their decider opted to transfer their MU to them. They further learned the group affiliation of their decider and the stage 1 decision of their decider. Note that in the instructions, it was explained to them that their receiver could not also be their decider in the same round. This was done to exclude the possibility of direct reciprocity. Following this feedback, the group moved to the next round starting with stage 1 again. To avoid reputation building, it was not possible for participants to identify or track behavior of other participants across rounds. After the main task, participants performed another task to measure trust and reciprocity within and across groups (*57*), individual-level social preferences (*58*), and identification with their group and the larger collective (*59*) and provided demographic information (see the Supplementary Materials for details). Screenshots of the instructions and computer interface are shown in figs. S7 to S17.

#### Experimental manipulation

Across groups, we manipulated whether participants would only be assigned a partner from their own group in stage 2 or not. Specifically, in the strict-boundary treatment (*N* = 21 groups), participants were only paired and interacted with a member from their own group (*p* = 1). In the fluid-boundary treatment (*N* = 21 groups), participants had the same likelihood to interact with an in-group or an out-group member (*p* = 0.5). Participants were informed about this rule in the instructions. Everything else was identical across these two treatments.

#### Payments

Participants received a participation fee of €5.50. In addition, one round was randomly chosen from the main task for payment, allowing them to earn an additional payment of maximally €7.63. In the additional tasks (see the Supplementary Materials), participants could further earn another €0.66. On average, participants earned €11 in the laboratory and £10.51 on Prolific. The experiment took around 1 hour in the laboratory and around 80 minutes on the Prolific platform (because of the longer waiting times until all participants joined a session and longer time that participants spent on instructions and comprehension questions).

#### Analyses

Data were analyzed using multilevel logistic regression models (as preregistered), because our dependent variables are binary (e.g., helping versus not helping or cooperating universally versus not), and data are nested within participants and groups. The models estimated two hierarchically nested intercepts (for each individual and for each group), a random slope (changes across rounds per group and individual), and the covariance across all random effects. Models were fitted using the lme4 package in R and applying the Satterthwaite degrees of freedom method to derive *P* values.

### Agent-based simulations

We used agent-based simulations to investigate larger populations, group sizes, and how a population split into multiple groups influences the evolution of universal cooperation. In all of these cases, the state space increases to such an extent that exact numerical simulations become unviable. To illustrate, the transition matrix of our numerical simulations entails 225 possible states for a given parameter combination, which increases to 1,758,276 possible states for *N* = 100, *k* = 2 and over 10^8^ for *N* = 100, *k* = 3. In the reported agent-based simulations, we investigated *k* = 8 groups with *n* = [4, 8, 16, 32, 64] agents per group (effect of group size) or a population of *N* = 128 agents split into *k* = [2, 4, 8, 16, 32] groups (effect of fragmentation) with *c*_c_ = *e* = *c*_h_ = 1, *b*_CG_ = 2, *b*_PG_ = 3, μ = 10^−4^, *p* = [0.5, 0.55, 0.6, …, 1.0], and *b*_h_ = [1, 1.2, 1.4, …, 4]. For each parameter combination, we collected 200 runs per manipulation, leading to a total of 352,000 independent simulations.

Agent-based simulations followed the exact rules of the numerical simulations. The composition of the population was initialized randomly at the start of each simulation run. In each iteration, agents accrued payoffs in stage 1 and stage 2, after which one agent was randomly selected to adapt its strategy based on random mutation (with probability μ) or imitation (with probability 1 − μ). In stage 2, each agent (in the role of the helper) was randomly paired with another agent (in the role of the receiver) from either the same or the other group, depending on *p*. The simulations were run until a homogeneous state was reached (i.e., a state in which all agents were of the same type) or for a maximum of 2000 iterations (in which case, we calculated the average population composition of the last 1000 runs). On average, simulations converged to a homogeneous state after 1351 iterations. Note that for the agent-based simulations, we extended the parameter space of *p* and also investigated *p* values lower than 0.5 (i.e., in the range from [1.0, …, 0.0]). On the basis of our definition, *p* determines the frequency with which in-group members interact with out-group members. Hence, *p* = 0.5 can be interpreted as group members having half of their interactions with in-group members. However, it is also possible that agents have more interactions with out-group members (i.e., spend more “time” outside their group), especially when populations are comprised of many groups.

In the Supplementary Materials, we report additional results varying *N* and *k* while fixing *n* (effect of the number of groups). We further introduce additional strategy types to investigate second-order free-riding.

## Supplementary Materials

### This PDF file includes:

Analytical results Numerical simulations Behavioral study Agent-based simulations Instructions and computer interface of the behavioral study Figs. S1 to S17 Tables S1 to S7 References
