# Supplementary material for: The evolution of universal cooperation
Source: Sci Adv. 2023 Feb 17;9(7):eadd8289. doi: 10.1126/sciadv.add8289 (PMC9937576; doi:10.1126/sciadv.add8289)
Supplement: Supplementary file 1 — Analytical results Numerical simulations Behavioral study Agent-based simulations Instructions and computer interface of the behavioral study Figs. S1 to S17 Tables S1 to S7 References [file sciadv.add8289_sm.pdf]

Science Advances
AAAS

Supplementary Materials for

The evolution of universal cooperation

Jörg Gross *et al.*

Corresponding author: Jörg Gross, mail@joerg-gross.net

Sci. Adv. **9**, eadd8289 (2023)
DOI: 10.1126/sciadv.add8289

This PDF file includes:

Analytical results
Numerical simulations
Behavioral study
Agent-based simulations
Instructions and computer interface of the behavioral study
Figs. S1 to S17
Tables S1 to S7
References

1. Analytical results

Recall that the parameter p stands for the probability of meeting an in-group partner in the dyadic interaction. Based on the expected payoffs of our three types of agents (see numerical simulations in the *Methods* section), we can characterize the population compositions at which various types will perform better or worse than others. To simplify the analysis, we will compare earnings of agents of different types that belong to the same group.

Universal cooperators will out-compete free riders as long as:

$$\pi_{Ui} > \pi_{Fi} \iff \left(p\frac{n_{Ui}-1}{n_i-1} + (1-p)\frac{n_U - n_{Ui}}{N-n_i}\right)(b_h - c_h) > c_c.$$

Similarly, we can compare parochial types with free-riders:

$$\pi_{Pi} > \pi_{Fi} \iff \left(p\frac{n_{Pi} - 1}{n_i - 1}\right)(b_h - c_h) > c_c,$$

As well as universal and parochial cooperators with each other:

$$\pi_{Ui} > \pi_{Pi} \iff p\frac{n_{Ui}-1}{n_i-1} + (1-p)\frac{n_U - n_{Ui}}{N-n_i} - p\frac{n_{Pi}-1}{n_i-1} > 0.$$

Recall the identity $N = kn_i$. We will use the following approximations:

$$\frac{n_{Ui}-1}{n_i-1} \sim \frac{n_{Ui}}{n_i} \text{ and } \frac{n_{Pi}-1}{n_p-1} \sim \frac{n_{Pi}}{n_i}.$$

We are mainly interested in the effect of p on the survival of each type. To examine whether free-riding prevails or disappears, we combine universalists and parochialists into a single category of cooperators.

Non-fluid groups: p = 1

$$\pi_{Ci} > \pi_{Fi} \Leftrightarrow \frac{\max(n_{Ui}, n_{Pi})}{n_i} > \frac{c_C}{b_h - c_h}.$$

This means that with non-fluid groups, the success of some form of cooperation requires that the share of either type of cooperator – whichever is the largest – in the group to exceed the costs-benefit ratio of cooperation-to-helping. This has the obvious implication that in our setting of $c_C = 1$, the net benefits of helping have to be at least 1.

Comparing the two forms of cooperation, we get:

$$\pi_{Pi} > \pi_{Fi} \Leftrightarrow n_{Ui} > n_{Pi}.$$

Thus, in a non-fluid case, the more numerous type of cooperator will spread.

Fluid groups: p = 0.5

$$\pi_{Ci} > \pi_{Fi} \Leftrightarrow \max((k-2)n_{Ui} + n_U, (k-1)n_{Pi}) > 2(k-1)n_i \frac{c_C}{b_h - c_h}.$$

The case of two groups ($k = 2$) provides an interesting special case. The condition becomes:

$$\pi_{Ci} > \pi_{Fi} \Leftrightarrow \frac{\max(n_U, n_{Pi})}{N} > \frac{c_C}{b_h - c_h}.$$

Effectively, the condition states that free-riders will be driven out if the share of either the *total* number of universal cooperators, or the share of in-group parochialists *in the entire population* exceeds the cost-benefit ratio of cooperation-to-helping. Compared to non-fluid groups, we see that out-group universalists begin playing a role in tipping the balance in favor of cooperation.

Comparing the two forms of cooperation, we get:

$$\pi_{Pi} > \pi_{Fi} \Leftrightarrow \frac{(k-2)n_{Ui} + n_U}{k-1} > n_{Pi}.$$

The left-hand side can be understood as a weighted average of in- and out-group universalists. For $k > 2$, we see that in-group universalists still receive a large weight. For $k = 2$, the condition simplifies to:

$$\pi_{Pi} > \pi_{Fi} \Leftrightarrow n_U > n_{Pi}.$$

Thus, the total number of universalists is pitted against only the in-group parochialists. If half of the population becomes universal cooperator, this condition is always satisfied. Hence, universalists are more likely to take over the population in groups with fluid, rather than non-fluid group boundaries.

Exploitation of universal cooperation

The above comparisons hold between agents of various types within the same group. However, and especially when considering competition between universal and parochial cooperators, parochial types may spread more easily. To understand this dynamic, consider the simple case of two groups, one composed entirely of universalists and the other entirely by parochialists. Parochialist types benefit from both their club good, as well as the public good paid for by universalists and will, hence, earn a higher payoff, and eventually drive out universalism.

Take the setting with two groups (k = 2), where one group is composed entirely of universalists, while the other contains both universalists and parochialists. Then, independently of fluidity p, parochial types can still dominate as long as:

$$\pi_{Pi} > \pi_{Uj} \Leftrightarrow \frac{n_{Pi}}{n_i} > \frac{b_h - c_h}{b_h - c_h + b_{CG}},$$

and

$$\pi_{Pi} > \pi_{Ui} \Leftrightarrow n_{Pi} > n_{Ui}.$$

Interestingly, in this setup, the competition between the two cooperative types is decided entirely by the costs and benefits of helping, as well as the return of the club good, and is independent of both fluidity and group size.

2. Numerical simulations

2.1. Imitation and contact

In our main model, we assume that imitation takes place across groups based on the payoffs of different strategies. The more successful a strategy is in the population, the more likely it is to be copied. However, one could also assume that group membership and the probability to meet in-group vs. out-group members (i.e., p) biases the strategy imitation process, such that an agent that

adapts their strategy has a higher likelihood to copy a strategy from an in-group member, the more likely it is to interact with in-group members in stage 2. In the extreme case, when in-group members only meet in-group members in stage 2 ($p = 1$), this would mean that agents only imitate strategies from the in-group, since they have 'no contact' with out-group members in stage 2. To test how these assumptions would influence the results of our model, we ran additional numerical simulations in which the probability to switch to a type y depends on the payoffs of type y in group A and B weighted by the probability of meeting in-group vs. out-group agents in the population:

$$p(x_A \to y) = \frac{p \times n_{sy_A} e^{\pi_{syA}} + (1-p) \times n_{sy_B} e^{\pi_{syB}}}{p(\sum_{t=1}^{l} n_{t_A} e^{\pi_{tA}}) + (1-p)(\sum_{t=1}^{l} n_{t_B} e^{\pi_{tB}})}$$

Under $p = 0.5$, the probability of agent x_A switching to strategy y is equally influenced by the relative success of strategy y in its own or the other group, exactly like in our original model. At the other extreme, under $p = 1$, the probability of an agent x_A to switch to strategy y only depends on the success of strategy y in group A. In other words, agents only imitate other strategies from their own group but are unaffected by the success of different strategies in the other group in this case. This corresponds to assuming that groups are completely isolated, and evolution (or learning) only takes place within each confined group (when $p = 1$). With decreasing p, the success of strategies in the opposing group are gradually integrated. Under $p = 0.5$, imitation takes place across group boundaries without any group bias in the learning process. Figure S1 shows the result of the numerical simulation for this adjusted Moran process.

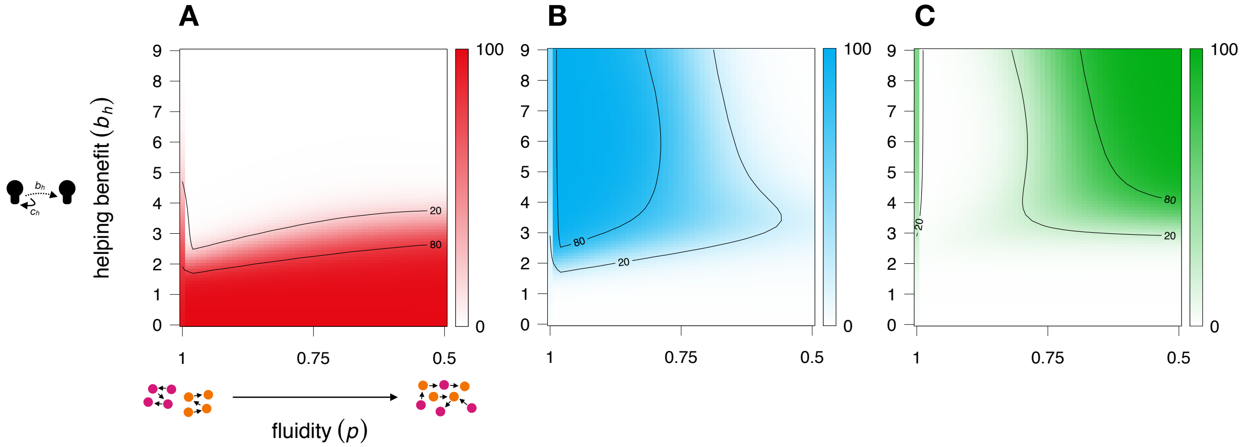

Figure S1. Numerical simulation results when the Moran process is biased by group membership and the probability to only interact with in-group members (p).

The general results are very similar to our original model specification (with 'global' learning; see Fig. 2A-C in the main manuscript) under $0.5 < p < 1$, and – by necessity – exactly the same under $p = 0.5$. Yet, under $p = 1$, we observe a similar proportion of universalists and parochialists under sufficiently high b_h. For example, under $b_h = 5$, four population compositions are stable, and they all emerge with the same probability of 17.7%: (a) a population of only parochialists, (b) a population of only universalists, (c) group A consisting of only parochialists and group B consisting of only universalists, (d) group A consisting of only universalists and group B consisting of only parochialists.

With this model setup, state (c) and (d) can be stable because there is no selection across groups taking place anymore. Parochialists in one group benefit from their club good and the public good provided for by universalists in the other group, leading to payoff inequalities across groups. Despite this inequality, universalists do not adopt their strategy in this case (or die out), since imitation is only taking place within the boundaries of the group. Hence, while groups are structurally interdependent (i.e., they share a public good), an assumption of complete isolation (i.e., no adaptation taking place across group boundaries) can, in this case, lead to (stable) cross-group exploitation.

The assumption of no learning across group boundaries under $p = 1$ thus creates a step transition in which certain configurations become stable that are not stable anymore already for slightly lower values of p. At the same time, for lower values of p, this new model does not generate qualitatively different results or insights.

2.2. Marginal per capita return (MPCR) of club goods and public good

In the main manuscript, we report how the parameters b_h and p influence the cooperation dynamics based on numerical simulations. Specifically, the results show how the benefit of helping (b_h) governs whether cooperating agents can survive against free-riders at all while the meeting probability within vs. between group boundaries (p) determines what type of cooperation emerges (i.e., universal or parochial cooperation; see also above). Another factor that influences the relative payoff of parochial vs. universal cooperators is the benefit from the club good (b_{CG}) relative to the benefit from the public good (b_{PG}) (*9–11*). In the reported simulations and the behavioural study, we set b_{CG} to 2 and b_{PG} to 3 (for the case of 2 groups with 4 agents each). This is an interesting case to consider because the individual return from universal cooperation is lower than the

individual return from parochial cooperation ($b_{PG}/N < b_{CG}/n$), while the efficiency (or, in other words, the social welfare of full cooperation) of public good provision is larger than club good provision (i.e., $b_{PG} > b_{CG}$).

Here we report additional results from numerical simulations in which we varied the parameters b_{PG} and b_{CG} along our main variables of interest (b_h and p). Figure S2 shows how these parameters influence the relative frequency of parochial vs. universal cooperation. With higher b_{CG}, parochial cooperation increasingly dominates the parameter space even when the likelihood to meet out-group members is high (i.e., low values of p; as can be seen in the change of the population composition from left to right in Fig. S2). Also note that this dynamic is unaffected by a change in b_{PG} (as can be seen by comparing the population composition from top to bottom in Fig. S2). This is because the benefits of public goods provision affect the payoff of all agents in the population equally by definition and cannot create a relative advantage for universal cooperators compared to other agents. In contrast, the benefit of club good cooperation provides a relative advantage for groups populated by parochialists compared to out-groups comprised of other types. This means that agents not only need to interact across group boundaries, but the relative return from local, group-confined club goods needs to be sufficiently low for universal cooperation to evolve.

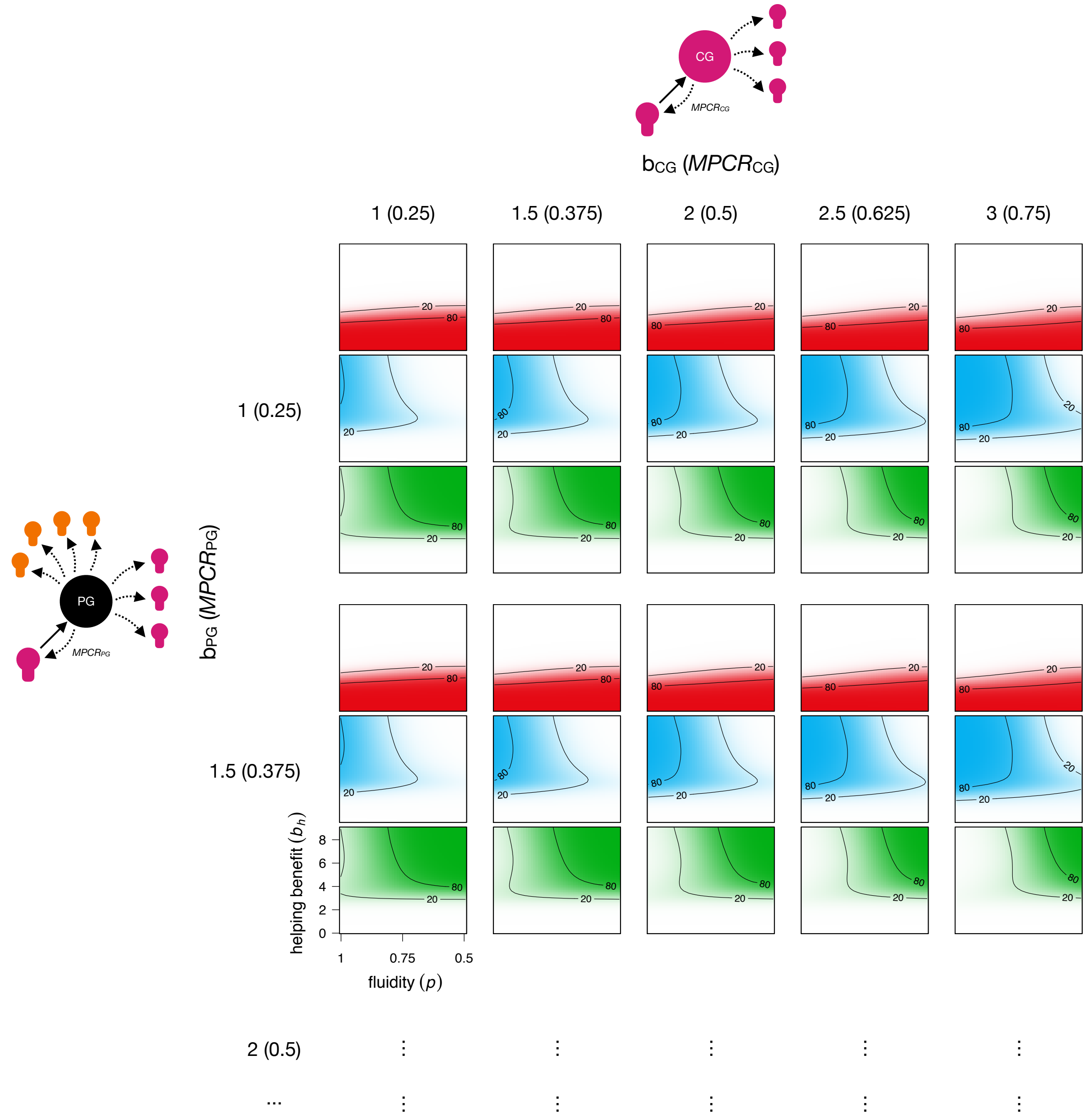

Figure S2. Evolution of universal cooperation depending on the return from club good cooperation. Relative frequency of free-riders (red), parochialists (blue), and universalists (green) depending on the benefit of club good cooperation (b_{CG}; varied along the rows) and the benefit of public good cooperation (b_{PG}; varied along the columns). The marginal per capita return (MPCR) is defined as the return from cooperation minus the cost of cooperation for a single agent and is simply calculated as b_{CG}/n and b_{PG}/N for the club good and public good, respectively, with $n = 4$ and $N = 8$. With a higher MPCR for club goods, it becomes more difficult to establish universal cooperation.

3. Behavioural study

3.1. Helping models

Our main helping model to test hypothesis 2 is shown in Table S1. As can be seen, participants help free-riders significantly less independent of the treatment. Importantly, universal cooperators are helped significantly more than parochial cooperators in the fluid compared to the solid group boundary treatment (fluid-boundary × universal receiver interaction), in line with our second hypothesis.

Table S1. Helping changes across treatments.

Fixed effects of a multilevel logistic regression predicting helping based on the receivers' cooperation decision in stage 1 in interaction with the treatment.

**Coefficient**	**est. (std. error)**	**p-value**
intercept (parochial receiver)	2.391 (0.363)	< .001***
selfish receiver	-1.517 (0.178)	< .001***
universal receiver	0.117 (0.142)	.412
fluid boundary treatment	-1.011 (0.515)	.0495*
fluid boundary × selfish receiver	-0.052 (0.255)	.838
fluid boundary × universal receiver	0.722 (0.198)	< .001***

Note: * p < .05, *** p < .001

We fitted additional models to investigate (1) to which degree helping decisions are influenced by own cooperation behavior, (2) to which degree participants conditioned their helping decisions on whether the receiver extended help in the previous round (i.e., second-order reputation information) and whether helping decisions were influenced by group affiliation (in the fluid group boundary treatment).

Table S2 shows that free-riders (i.e., participants deciding to keep their resources in stage 1) were significantly less likely to extend help in stage 2, resonating with our assumptions about free-riding agents in the simulations. Overall, participants who cooperated in stage 1 (either universally or parochially) decided to help their receiver in 78.6% of the cases which dropped by 40 percentage

points for participants who decided to keep their unit and did not cooperate in the cooperation stage. The likelihood of universal cooperators to help was not significantly different from that of parochial cooperators. Furthermore, focusing on participants' own cooperation decisions in interaction with the cooperation decision of the receiver, universal cooperators were more likely to help other universal cooperators compared to parochial cooperators (b = 0.50, p = .02).

Table S2. Helping as a function of own stage 1 decisions.

Fixed effects of a multilevel logistic regression predicting helping based on own cooperation decisions in stage 1.

**coefficient**	**est. (std. error)**	**p-value**
intercept (parochial cooperation)	2.106 (0.351)	< .001***
keep	-0.834 (0.186)	< .001***
universal cooperation	0.163 (0.142)	.252
fluid boundary treatment	-0.541 (0.499)	.278
fluid boundary × keep	-0.197 (0.266)	.458
fluid boundary × universal cooperation	0.026 (0.200)	.898

Note. *** p < .001

In Table S3, we see that participants in the solid-boundary treatment extended more help in stage 2 towards parochial and universal cooperators compared to free-riding participants (as already seen in Table S1 across the entire sample). Furthermore, participants were sensitive to second-order reputation information. The odds to help increased by 4.5 when the receiver was also a helper in the previous round.

Table S3. Helping as a function of receiver's decisions in the solid-boundary treatment.

Fixed effects of a multilevel logistic regression predicting helping based on the receiver's choice in stage 1 and helping decision in stage 2 (of the previous round) in the solid-boundary treatment.

	Model 1	Model 2
**coefficient**	**est. (std. err)**	**est. (std. err)**
intercept (parochial receiver)	2.51 (0.40)***	1.20 (0.34)***
selfish receiver	-1.56 (0.18)***	-1.42 (0.19)***
universal receiver	0.09 (0.14)	0.09 (0.15)
receiver helped (t-1)		1.64 (0.14)***

Note. *** p < .001

Finally, Table S4 shows how participants conditionally helped in the fluid-boundary treatment. As in the solid-boundary treatment, participants were significantly less likely to help receivers that decided to keep their unit in the previous cooperation stage and more likely to help receivers that also helped in the previous round. Furthermore, participants were more likely to help universal cooperators compared to parochial cooperators (in line with the model results shown in Table S1). This was partly driven by group affiliation. Participants significantly reduced their helping when paired with a parochial cooperator from the out-group (out-group receiver coefficient in Table S4, model 5). In contrast, their odds of helping did not significantly change when facing a universal receiver of the in-group or the out-group (post-hoc comparison: out-group receiver + universal × out-group receiver = 0; $b = -0.21$, $p = .16$), resonating with our model assumptions.

Table S4. Helping as a function of receiver's decisions in the fluid-boundary treatment.

Fixed effects of a multilevel logistic regression predicting helping based on the receiver's choice in stage 1, helping decision in stage 2 (of the previous round), and group affiliation in the fluid-boundary treatment.

	**Model 1**	**Model 2**	**Model 3**	**Model 4**	**Model 5**
**coefficient**	**est. (std. err)**	**est. (std. err)**	**est. (std. err)**	**est. (std. err)**	**est. (std. err)**
intercept (parochial receiver)	1.31 (0.33)***	0.35 (0.30)	0.23 (0.33)	0.55 (0.31)†	0.79 (0.34)*
selfish receiver	-1.52 (0.18)***	-1.36 (0.19)***	-1.23 (0.29)***	-1.37 (0.19)***	-1.65 (0.30) ***
universal receiver	0.77 (0.13)***	0.69 (0.15)***	0.86 (0.23)***	0.69 (0.15)***	0.38 (0.23)
receiver helped (t-1)		1.51 (0.13)***	1.71 (0.25)***	1.52 (0.13)***	1.52 (0.13)***
out-group receiver				-0.32 (0.11)**	-0.72 (0.25)**
selfish × out-group receiver					0.47 (0.39)
universal × out-group receiver					0.51 (0.29)†

Note. † $p < .10$, * $p < .05$, ** $p < .01$, *** $p < .001$

3.2 Additional measures

After the main task, we measured individual-level social preferences, self-reported identification, and a within/between group trust game in 304 participants in our sample (as pre-registered at https://aspredicted.org/VY8_C51). We did not pre-register hypotheses for these additional measures, and only included them for exploratory purposes. Results for these additional tasks are reported below.

3.2.1 Social preferences

We measured individual level social preferences using the 6-item social value orientation (SVO) slider measure (*58*). In this task, participants have to decide how to distribute points between themselves and another unknown person. For example, the participant has to choose one out of nine possible allocations ranging from allocating 100 points to oneself and 50 points to the other person (maximally 'pro-self' option) to allocating 50 points to oneself and 100 points to the other person (maximally 'pro-social' option). The decision pattern of participants allows to calculate a single measure of social preferences: the SVO angle. The higher the SVO angle, the more a person is willing to sacrifice points in order to benefit another person (i.e., higher SVO angle indicates

stronger social preferences). As such, decisions are altruistic in the sense that pro-social actions cannot be ascribed to reciprocity concerns (since they act with an anonymous other that cannot reciprocate kind actions).

We used the individual level SVO scores to further investigate decisions to contribute towards the public good, club good, or keep resources. Since we only had one SVO data point per individual, we aggregated data across rounds and fitted multilevel linear regression models to the average universal, parochial, and keeping decisions with a random intercept to account for individuals being nested in groups. A higher SVO angle was associated with less free-riding (i.e., keeping) decisions, as one would expect (Table S5). Further, participants with stronger social preferences contributed more to the public good (Table S6), independent of the treatment (see also (*3*) for similar findings). Interestingly, social preferences did not significantly predict the individual's propensity to cooperate with the in-group (i.e., club good provision, Table S7), also not in interaction with the treatment. Figure S3 illustrates the bivariate correlations between social preferences and contribution decisions.

Table S5. Social preferences and free-riding.

Multilevel regression modelling the average degree of keeping units as a function of individual's social preferences and treatment.

**coefficient**	**est. (std. error)**	**p-value**
intercept	0.314 (0.047)	< .001***
SVO angle	-0.006 (0.001)	< .001***
strict boundary treatment	-0.037 (0.067)	.575
SVO angle × strict-boundary	0.002 (0.002)	.331

Note. *** p < .001

Table S6. Social preferences and universal cooperation.

Multilevel regression modelling contributions to the public good as a function of individual's social preferences and treatment.

coefficient	est. (std. error)	p-value
intercept	0.437 (0.066)	< .001***
SVO angle	0.007 (0.002)	< .001***
strict boundary treatment	-0.069 (0.092)	.457
SVO angle × strict-boundary	-0.002 (0.002)	.358

Note. *** p < .001

Table S7. Social preferences and parochial cooperation.

Multilevel regression modelling contributions to the club good as a function of individual's social preferences and treatment.

coefficient	est. (std. error)	p-value
intercept	0.250 (0.053)	< .001***
SVO angle	-0.001 (0.001)	.450
strict boundary treatment	0.110 (0.075)	.143
SVO angle × strict-boundary	0.000 (0.002)	.955

Note. *** p < .001

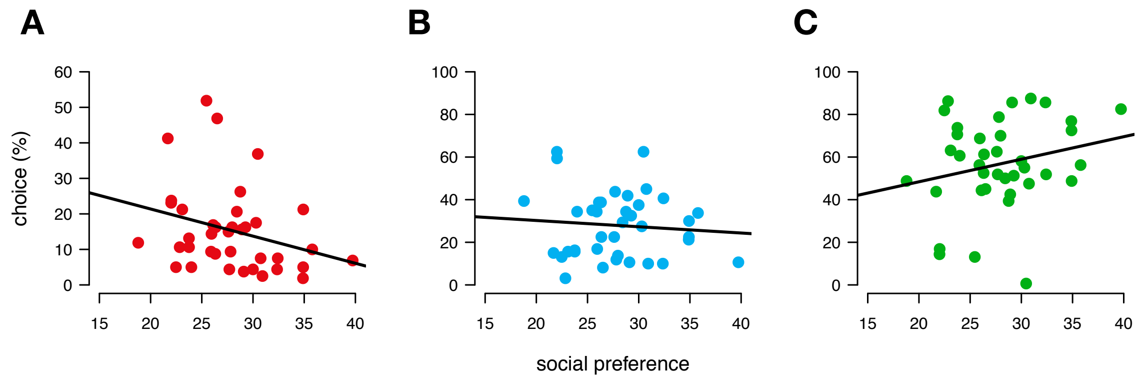

Figure S3. Social preferences and contribution decisions. Participants with higher social preferences (as measured by the SVO angle) were less likely to keep their resources (Spearman r = -0.31; **A**) and more likely to contribute resources to the public good (Spearman r = -0.29; **C**), while there was no significant association with parochial cooperation (Spearman r = -0.08; **B**). Each dot represents one participant. The black line indicates the best linear fit.

This suggests that social preferences modulate the likelihood of participants to choose universal cooperation rather than free-ride but are unrelated to parochial cooperation. This is important, because it can explain the high levels of universal cooperation in our experiment. According to this result, social preferences shift human behavior from free-riding towards more public good cooperation in general (but not parochial cooperation). In some sense, social preferences can, thus, be interpreted as a mechanism to establish cross-group cooperation that acts as a psychological substitute for intergroup interactions.

We should note that 70.4% of participants in our sample were classified as 'pro-social' (with 28.9% as 'selfish' and 1 participant as 'competitive' and 1 participant as 'altruistic') according to the SVO task. These values are higher than we observed previously in our lab and in other studies (*55*, *56*, *60*). One reason may be that data was collected during the COVID-19 pandemic which may increase social concerns in general and, due to selection bias, the social concerns of those who voluntarily participate in scientific research, specifically. This, however, does not challenge our main conclusions about how inter-group interactions shift cooperation towards universal and away from parochial cooperation, since these conclusions rest on relative differences in universal vs. parochial cooperation across our experimental treatments and participants were randomly assigned to treatments independent of their social preferences. Indeed, we did not find a significant difference in social preferences across our two treatments (multilevel regression, treatment $b = 0.234$, $t(302) = 0.151$, $p = .88$).

3.2.2 Group identification

After experiencing the task, we asked participants to indicate their identification with their group of four participants with which they shared a club good (item 1: 'I felt a bond with my group', item 2: 'I am glad that I was in my group', item 3: 'I felt solidarity with my group', item 4: 'I felt committed to my group') and the larger collective (i.e., the eight person group that shared a public good; item 1: 'I felt a bond with all participants', item 2: 'I am glad that I was part of the larger collective', item 3: 'I felt solidarity with all participants', item 4: 'I felt committed to all participants') on a four-point Likert scale from 'not at all' to 'very strongly' (*59*).

The internal consistency of responses was Cronbach's $\alpha = 0.88$ [95% CI: 0.85–0.90] for the group identification scale and Cronbach's $\alpha = 0.88$ [95% CI: 0.86–0.90] for the cross-group identification scale. In other words, participants sufficiently answered individual items in a similar way to allow

aggregating responses across items and to calculate a scale mean for self-reported in-group identification and cross-group identification. For each individual, we calculated the difference between cross-group and in-group identification. Positive values indicate a higher cross-group identification and negative values indicating a higher in-group identification.

Figure S4 shows how the experimental manipulation influenced these self-reported identification scores. In the strict-boundary treatment (p = 1), participants indicated to identify as much with their in-group as with the larger collective with an average of $\bar{x}$ = -0.05 that did not significantly differ from zero (one-sample Mann-Whitney U-test, U = 94, p = .70). In the fluid-boundary treatment (p = 0.5), participants' difference scores were significantly above zero ($\bar{x}$ = 0.53; one-sample Mann-Whitney U-test, U = 168, p < .001) indicating that they identified more with the larger collective than with the in-group and significantly more so than in the strict-boundary treatment (Mann-Whitney U-test, U = 56, p < .001).

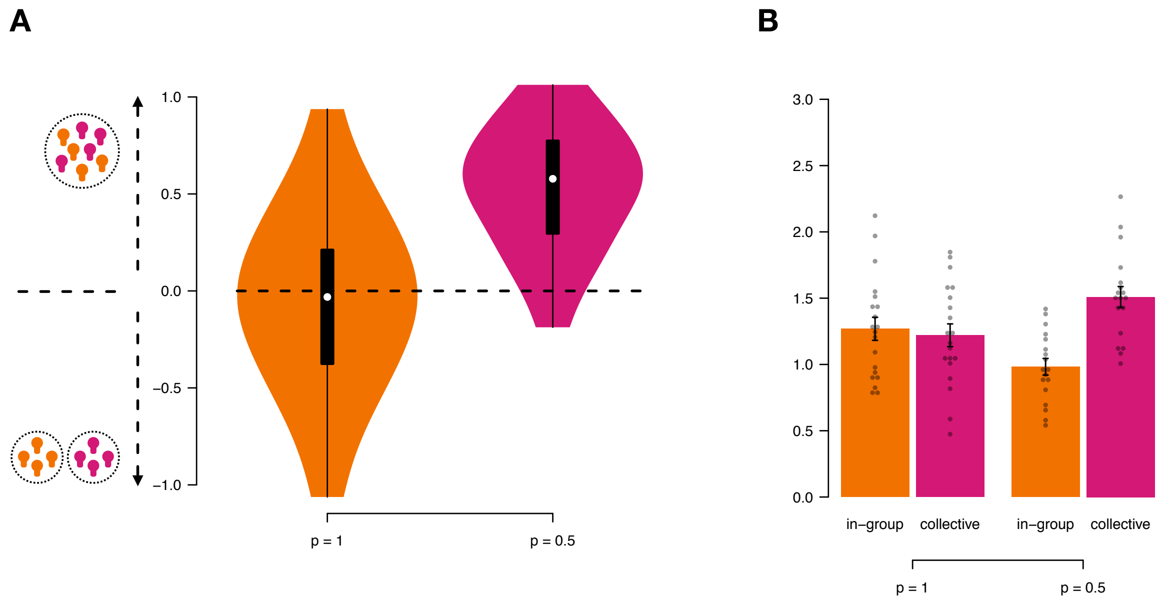

Figure S4. Self-reported identification. (**A**) Distribution of difference scores per individual calculated as the average identification across groups minus the average identification with the in-group in the strict-boundary (p = 1) and fluid-boundary (p = 0.5) treatment. Positive values indicate a stronger self-reported identification with the larger collective than the in-group. Results show that higher fluidity increases identification with the larger collective. (**B**) Average identification for the in-group and the collective separated by treatments further shows how self-reported identification with the in-group decreases in the fluid boundary treatment, while identification with the larger collective increases compared to the strict boundary treatment. White dots indicate the medians, black boxes the upper and lower quartiles, vertical lines the 95% intervals (for A), error bars indicate the standard error of the mean and dots represent averages per group (for B).

3.2.3 Trust

Immediately after finishing the main task, participants made trust- and reciprocity decisions in two trust games (*57*). In the two trust-decisions, participants received 5 units of endowment each and had to decide how many of these units to send to a receiver and how many of these units to keep for themselves. In the first trust decision, the receiver was a member from the own group. In the second trust decision the receiver was a member from the other group from the previously played nested social dilemma.

Participants were told that each transferred unit was multiplied by 3 and that the receiver could then decide how many of the received units to transfer back to them. The mutually most efficient and fairest outcome is achieved when all units are transferred (leading to 15 units and, hence, a surplus of 10 units) and the receiver transfers 7 or 8 units back to. Transfers to the receiver can be considered a measure of trust, since the risk of transferring units is that the receiver will not transfer anything (or less than what was transferred to them) back. Back-transfers of the receiver can be considered a measure of reciprocity. The more the receiver transfers back, the more she reciprocates the trust she received.

Reciprocity of trust was measured with the strategy method (see, e.g., (*18*)). For each possible transfer, the participant had to indicate how many units she wants to transfer back and how much she wants to keep for herself. Participants made this decision twice, for transfers by an in-group member and for transfers by an out-group member. Decisions had real payoff-consequences and participants were paired in a closed loop (subject 1 sending to subject 2, subject 2 to subject 3, …, subject 8 to subject 1) within and between groups such that no receiver was also the sender for of the same partner. This was known to participants to avoid the possibility of direct reciprocal interactions.

We tested whether participants extended or reciprocated more trust towards in- vs. out-group members in interaction with the treatment they experienced. In general, we found that participants transferred 0.5 units more to in-group members on average (multilevel regression, $b = 0.49$, $p < .001$). We did not find any evidence that the treatment affected average trust (towards in-group vs. out-group members) or reciprocity. However, we observed that higher universal cooperation significantly reduced the trust-gap between in-group and out-group members (multilevel regression, $b = -0.56$, $p = .04$).

4. Agent-based simulations

4.1 Group size effects on the evolution of universal cooperation

Our agent-based simulations extended the numerical simulations by investigating larger and more groups. The parameters governing the population structure are N (size of the population) and k (number of groups that exist in the population) with $n = N/k$ giving the number of agents per group. The simulations reported in the main manuscript investigated the trajectory of cooperation assuming a fixed k (number of groups) and manipulating N and n (Fig. 4A; group size effect) and assuming a fixed N (population size) and manipulating k and n (Fig. 4B, fragmentation effect). Here, we report results from additional simulations in which we fixed n (group size), manipulating k and N. Hence, we complement the already reported results and investigate the third possibility to manipulate population structure by varying the number of groups of equal sizes (i.e., a number of groups effect).

Figure S5 shows the emergence of parochial vs. universal cooperation when varying the number of groups k, fixing the group size n to 16 (leading to a population of size of N = [32, 64, 128, 256, 512]), respectively. As can be seen in Figure S5A, parochialism does not substantially increase when keeping the group size constant but increases the sharpness of the transition between parochialism and universalism (as illustrated Fig. S5B). This shows that a smaller vs. a larger number of groups per se is not detrimental for the emergence of universal cooperation but rather many small groups vs. few large groups (i.e., a higher fragmentation of a fixed-sized population). Results are based on 200 simulations for each parameter combination (176,000 independent simulations in total).

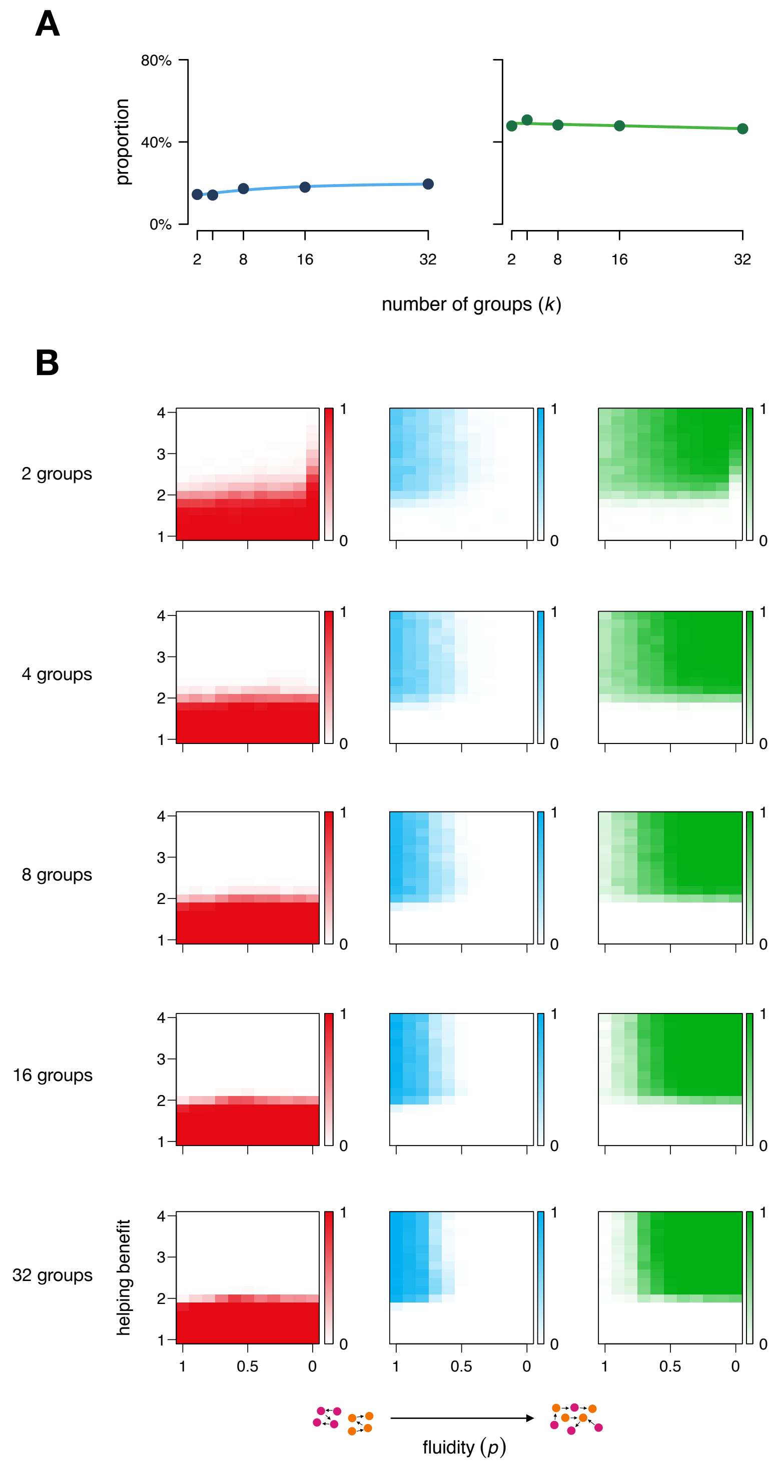

Figure S5. Increase in the number of groups. When populations consist of more groups (of equal size n = 16), the relative frequency of parochial cooperation (left) and universal cooperation (right) stays relatively constant (**A**); based on k = [2, 4, 8, 16, 32] groups, averaged across p = [1.0, …, 0.0] and b_h = [1, …, 4]. Across the whole parameter space (with $c_c = c_h = 1$, $b_{CG} = 2$, $b_{PG} = 3$, $\mu = 10^{-4}$), the overall frequency of parochialism to universalism does not change substantially, while the transition depending on p becomes sharper with more groups (**B**).

4.2 Conditional helping and second-order free-riding

In our simulations, we assume that agents help depending on the first stage behavior of their receiver. This reciprocity mechanism allows to exchange mutual benefits among cooperators and can reverse the payoff gap between free-riders and cooperators. Exchanging conditional help among cooperators is therefore (a) essential to outcompete free-riders and (b) governs what type of cooperation emerges, depending on p (since we assume that universalists help other universalists while parochialists help other parochialists of their own group, only).

To be able to derive exact numerical simulations, we had to restrict the type space to a small set of types that already condition help on first stage behavior (the receiver's cooperation choice) as well as the receiver's group affiliation. In this restricted type space we do not consider, for example, types that disregard group membership and/or promote cooperation in general (i.e., regardless of whether cooperation is parochial or universal). Furthermore, it is important to note that second stage helping is a costly action and therefore also introduces free-riding incentives and a second-order free-riding problem that is not captured in the original model. A non-helping cooperator (i.e., free-riding on the possibility to enforce a norm of cooperation) should outcompete a helping cooperator that is willing to pay the cost to reward cooperation.

To extend the strategy space and also incorporate this second-order free-rider problem, we ran additional agent-based simulations. In a first step, we introduce three helping types: helping universalists, helping parochialists and 'non-discriminating helpers' that reward both parochialists and universalists. Importantly, in this first step, agents help regardless of the group membership of the receiver and there are no cooperating agents that do not extend help (i.e., we do not consider second-order free-riders in this first step). As shown in Figure S6A, cooperation can evolve in this type space, as long as the benefit of helping is larger than 2. The type of cooperation (universalism vs. parochialism) can both emerge independent of p, since helping is not conditional on group affiliation. Importantly, while cooperation can stabilize and free-riding disappears, agents that help regardless of the type ('non-discriminating helpers') disappear in the population, being replaced by either exclusive universal helpers or parochial helpers (on average, only 0.4% of agents are 'non-discriminating helpers' at the end of the simulations vs. 30% universal and parochial helping types, respectively). This already shows that, even when helping is unconditional on group

affiliation, indiscriminate helping is driven out (since it is more costly than discriminatory helping).

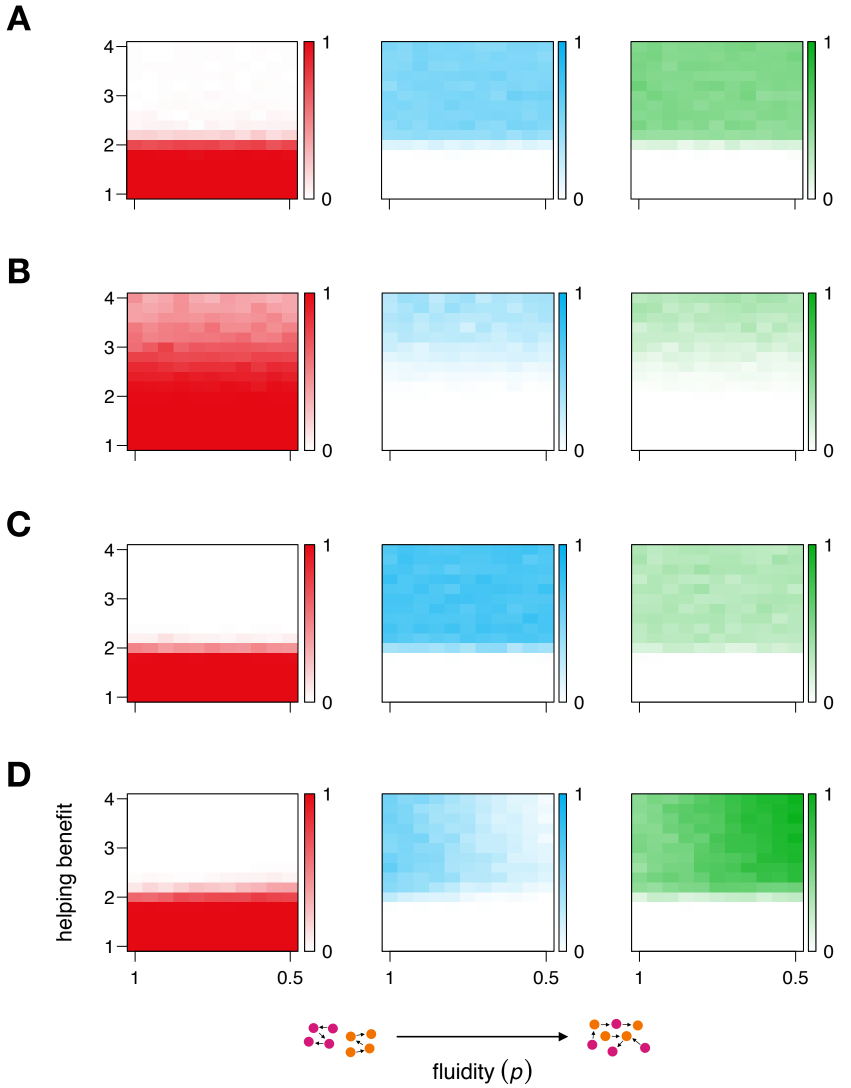

Figure S6. Extended type space and incorporating second-order free-riding. (**A**) With three helping types that cannot condition their helping on group affiliation, cooperation evolves with sufficient helping benefit, but the type of cooperation that emerges is unaffected by p. (**B**) When introducing the possibility for second-order free-riding, selfish agents have a higher chance to survive and take over the population, even under high helping benefit (b_h). (**C**) When agents condition their help only on stage 2 reputation information, parochial cooperation is more prevalent and the type of cooperation that emerges is unaffected by the fluidity of group boundaries (p). (**D**) However, when agents condition their helping on both stage 1 and stage 2 behavior, the same general pattern emerges again; i.e., increased fluidity of group boundaries increases universal and decreases parochial cooperation. Based on $c_c = c_h = 1$, $b_{CG} = 2$, $b_{PG} = 3$, $\mu = 10^{-4}$, $k = 2$, $n = 50$.

In the next step, we introduced second-order free-rider types – i.e., universal cooperators that do not extend help in stage 2 and parochial cooperators that do not extend help in stage 2 – next to our four original types (helping universalists, helping parochialists, 'non-discriminating helpers', and non-helping free-riders). We further assume, as before, that helping agents only condition their help on the stage 1 behavior of their receiver (i.e., whether they cooperate parochially, universally, or free-ride). Hence, there is no mechanism to 'punish' second-order free-riding. Figure S6B shows that under these circumstances, free-riders dominate the parameter space, because non-helping types have an advantage over helping types, while free-riders have an advantage over non-helping types. Second-order free-riding crowds out cooperation when agents cannot condition their help on the helping behavior of their receivers.

Therefore, helping types need a mechanism to identify and exclude non-helping types from receiving help (i.e., second stage reputation information). In the second model, we consider that agents can condition their stage 2 decision on whether the receiver is a helping type or not. Specifically, helping agents only help other agents when they also are willing to costly help in stage 2 (i.e., are also helping types). Importantly, this conditional help does not depend on stage 1 behavior. It only matters whether agents are a helping type or not, but not whether they are universal cooperators or parochial cooperators. This allows cooperation to emerge again. Yet, since conditional behavior is only conditional on stage 2, the fluidity of group boundaries (p) does not matter and we observe a higher proportion of parochial cooperation regardless of p as long as b_h is large enough (Fig. S6C).

For p to influence the type of cooperation that emerges, agents need to condition their help on a combination of stage 1 and stage 2 reputation. In other words, agents need not only care about whether their receiver is also willing to help, but also what type of cooperation they reward. In the last model, we therefore assume that helping agents condition their help on a combination of stage 1 and stage 2 behavior of their receiver. Specifically, next to free-riders, non-helping parochialists and non-helping universalists, we assume that helping universalists (or 'non-discriminating helpers') only extend help when their receiver is also a helper of other universalists and helping parochialists (or 'non-discriminating helpers') only extend help when their receiver also is a helper of other parochialists (and part of their own group). When agents combine reputation information of stage 1 and stage 2 in this way, the simulations show that cooperation likewise emerges again (Fig. S6D) and cooperating agents can alleviate the second-order free-rider problem. Importantly,

the fluidity of group boundaries influences the type of cooperation again in the direction that we observed in all of our reported theoretical models and in line with our empirical observations: With higher fluidity, universal cooperation emerges, whereas with lower fluidity of group boundaries, parochial cooperation is favored (as long as b_h is high enough; see Fig. S6D).

Importantly, our empirical data are in line with this overall pattern of cooperation (i.e., increases in parochial vs. universal cooperation depending on p), which suggests that participants are sensitive to reputation information from stage 1 and stage 2. Furthermore, looking at what information participants used to decide to help the receiver or not, we found empirical evidence that participants conditioned their helping both on first stage behavior (i.e., the cooperation decision of their receiver) and their second stage behavior (i.e., whether the receiver helped in the past or not – see section 3.1).

5. Instructions and computer interface of the behavioural study

Figure S7-S13 show the instructions as displayed on the computer of participants. Figure S14-S17 show the computer interface for decision making in the main experiment.

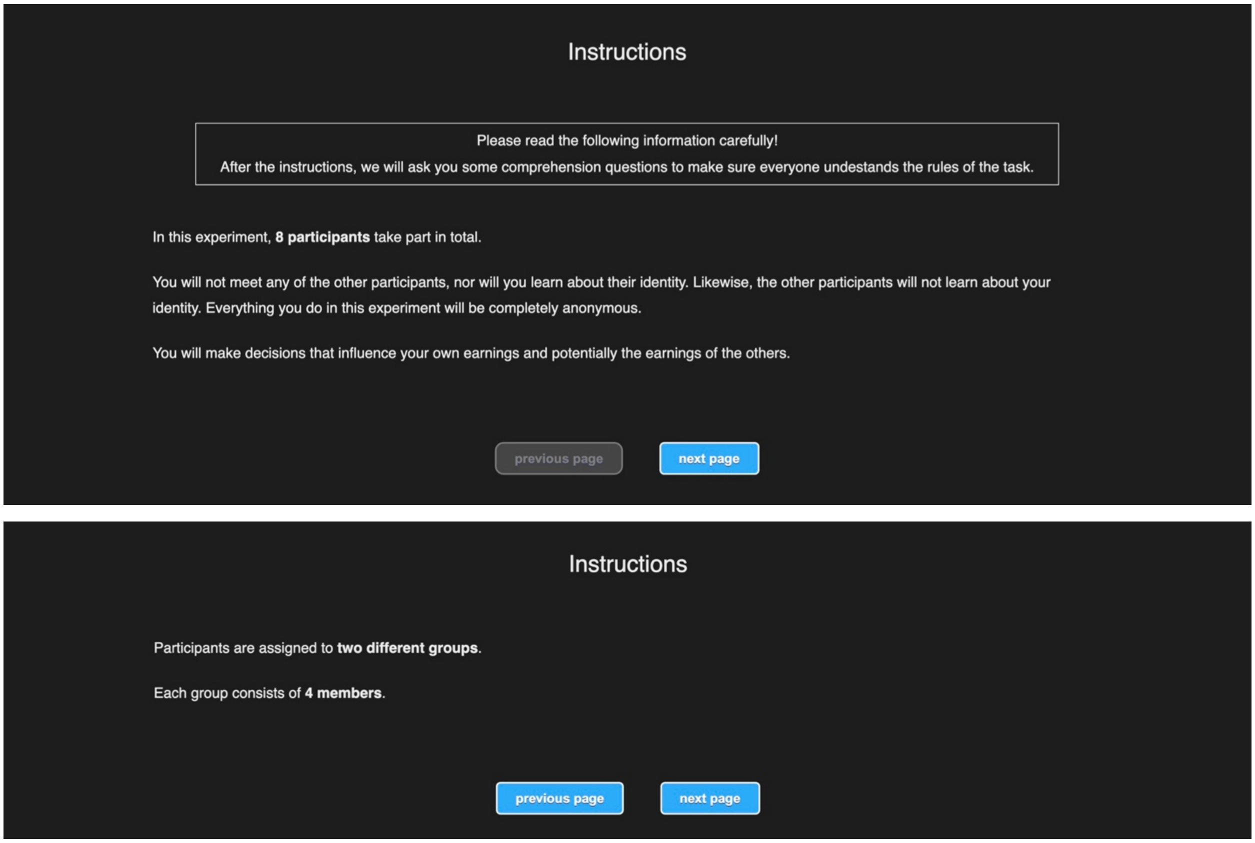

Figure S7. Instructions (page 1 and 2).

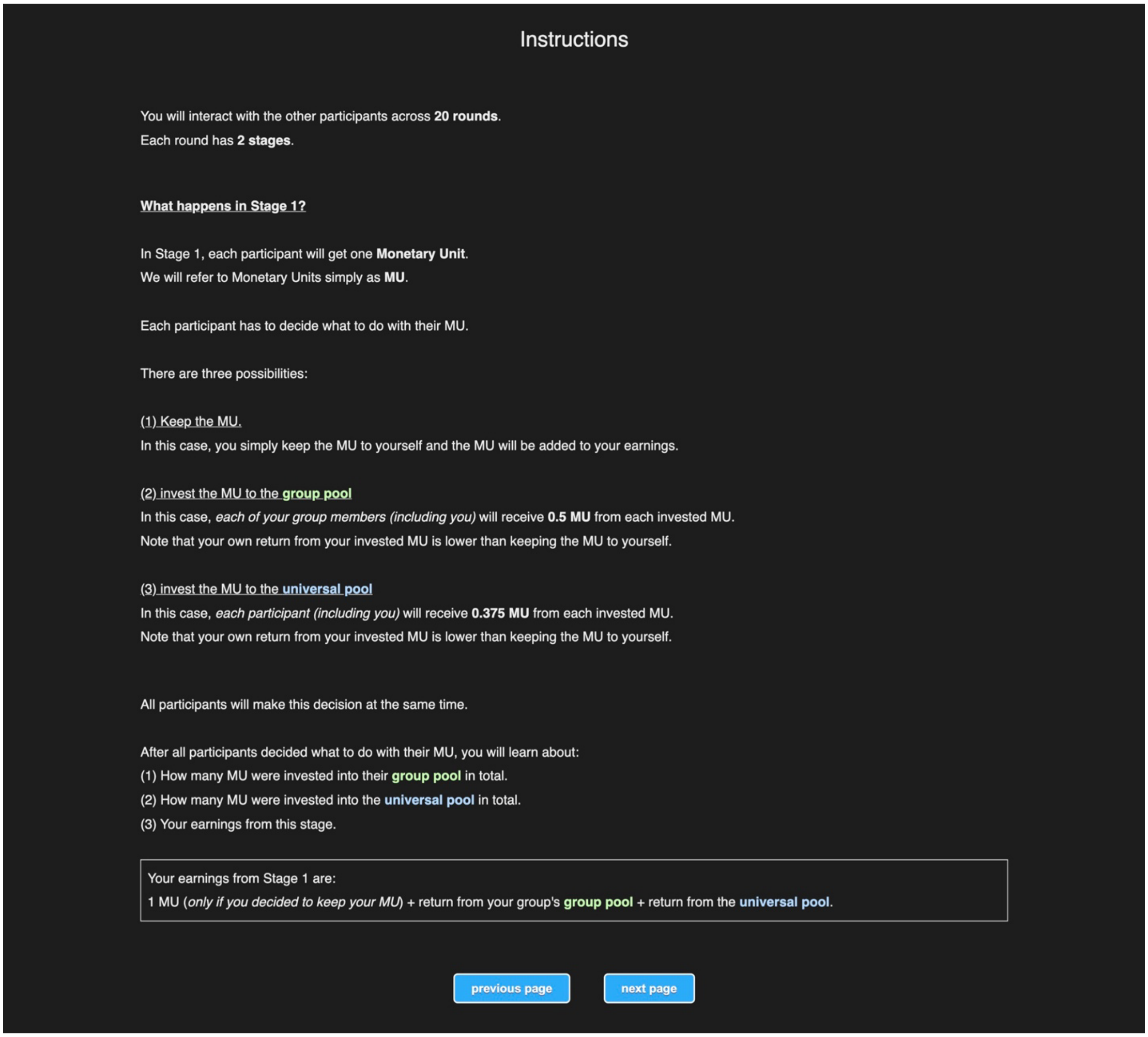

Instructions

You will interact with the other participants across **20 rounds**.
Each round has **2 stages**.

What happens in Stage 1?

In Stage 1, each participant will get one **Monetary Unit**.
We will refer to Monetary Units simply as **MU**.

Each participant has to decide what to do with their MU.

There are three possibilities:

(1) Keep the MU.
In this case, you simply keep the MU to yourself and the MU will be added to your earnings.

(2) invest the MU to the **group pool**
In this case, *each of your group members (including you)* will receive **0.5 MU** from each invested MU.
Note that your own return from your invested MU is lower than keeping the MU to yourself.

(3) invest the MU to the **universal pool**
In this case, *each participant (including you)* will receive **0.375 MU** from each invested MU.
Note that your own return from your invested MU is lower than keeping the MU to yourself.

All participants will make this decision at the same time.

After all participants decided what to do with their MU, you will learn about:
(1) How many MU were invested into their **group pool** in total.
(2) How many MU were invested into the **universal pool** in total.
(3) Your earnings from this stage.

Your earnings from Stage 1 are:
1 MU (*only if you decided to keep your MU*) + return from your group's **group pool** + return from the **universal pool**.

previous page | next page

Figure S8. Instructions for stage 1 (page 3).

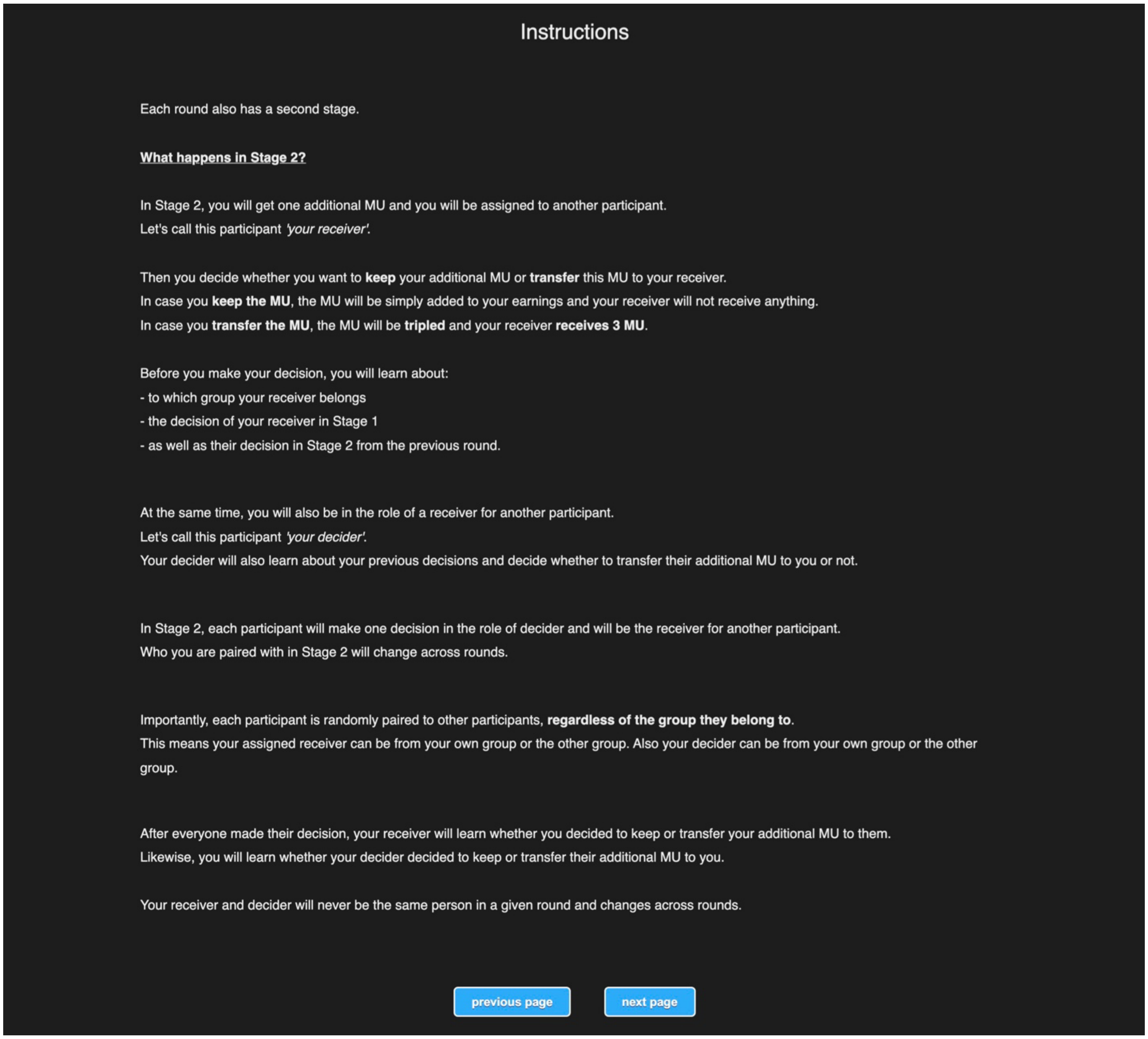

Instructions

Each round also has a second stage.

What happens in Stage 2?

In Stage 2, you will get one additional MU and you will be assigned to another participant.
Let's call this participant *'your receiver'*.

Then you decide whether you want to **keep** your additional MU or **transfer** this MU to your receiver.
In case you **keep the MU**, the MU will be simply added to your earnings and your receiver will not receive anything.
In case you **transfer the MU**, the MU will be **tripled** and your receiver **receives 3 MU**.

Before you make your decision, you will learn about:
- to which group your receiver belongs
- the decision of your receiver in Stage 1
- as well as their decision in Stage 2 from the previous round.

At the same time, you will also be in the role of a receiver for another participant.
Let's call this participant *'your decider'*.
Your decider will also learn about your previous decisions and decide whether to transfer their additional MU to you or not.

In Stage 2, each participant will make one decision in the role of decider and will be the receiver for another participant.
Who you are paired with in Stage 2 will change across rounds.

Importantly, each participant is randomly paired to other participants, **regardless of the group they belong to**.
This means your assigned receiver can be from your own group or the other group. Also your decider can be from your own group or the other group.

After everyone made their decision, your receiver will learn whether you decided to keep or transfer your additional MU to them.
Likewise, you will learn whether your decider decided to keep or transfer their additional MU to you.

Your receiver and decider will never be the same person in a given round and changes across rounds.

previous page | next page

Figure S9. Instructions for stage 2 (page 4).

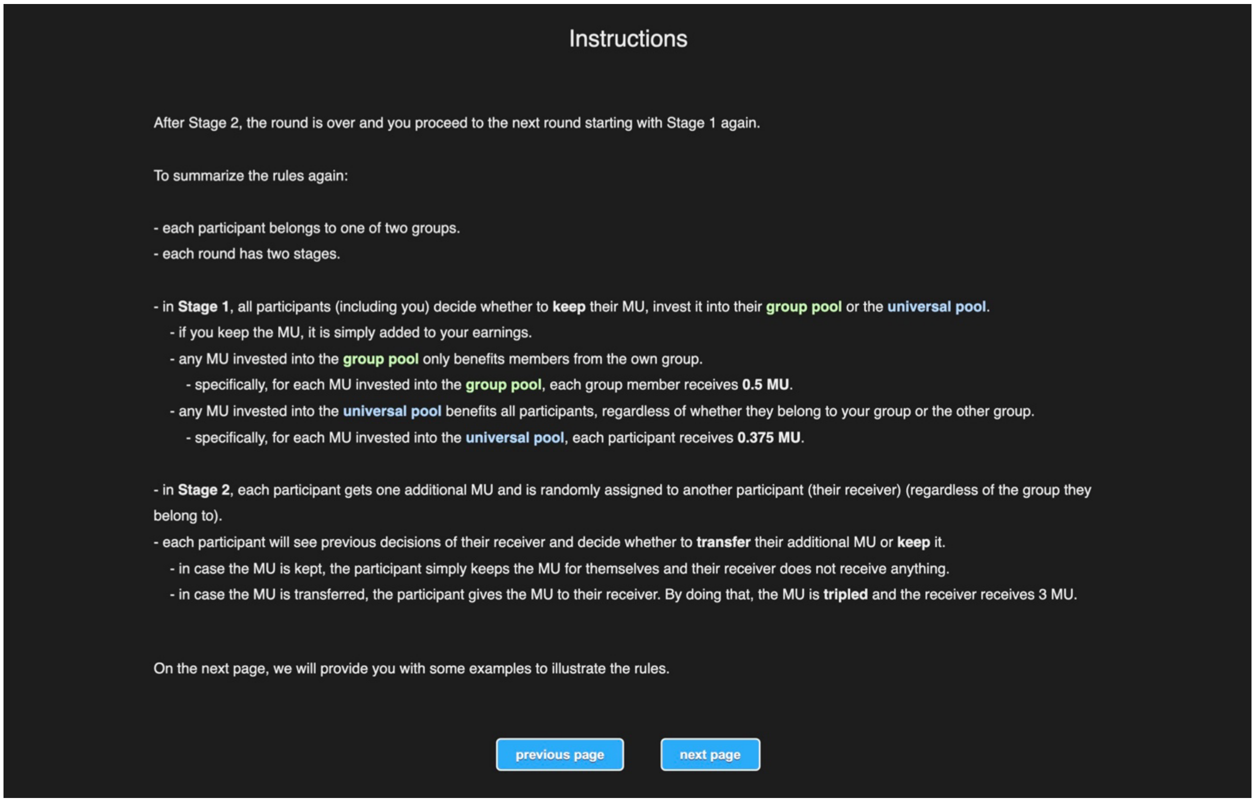

Figure S10. Summary (page 5).

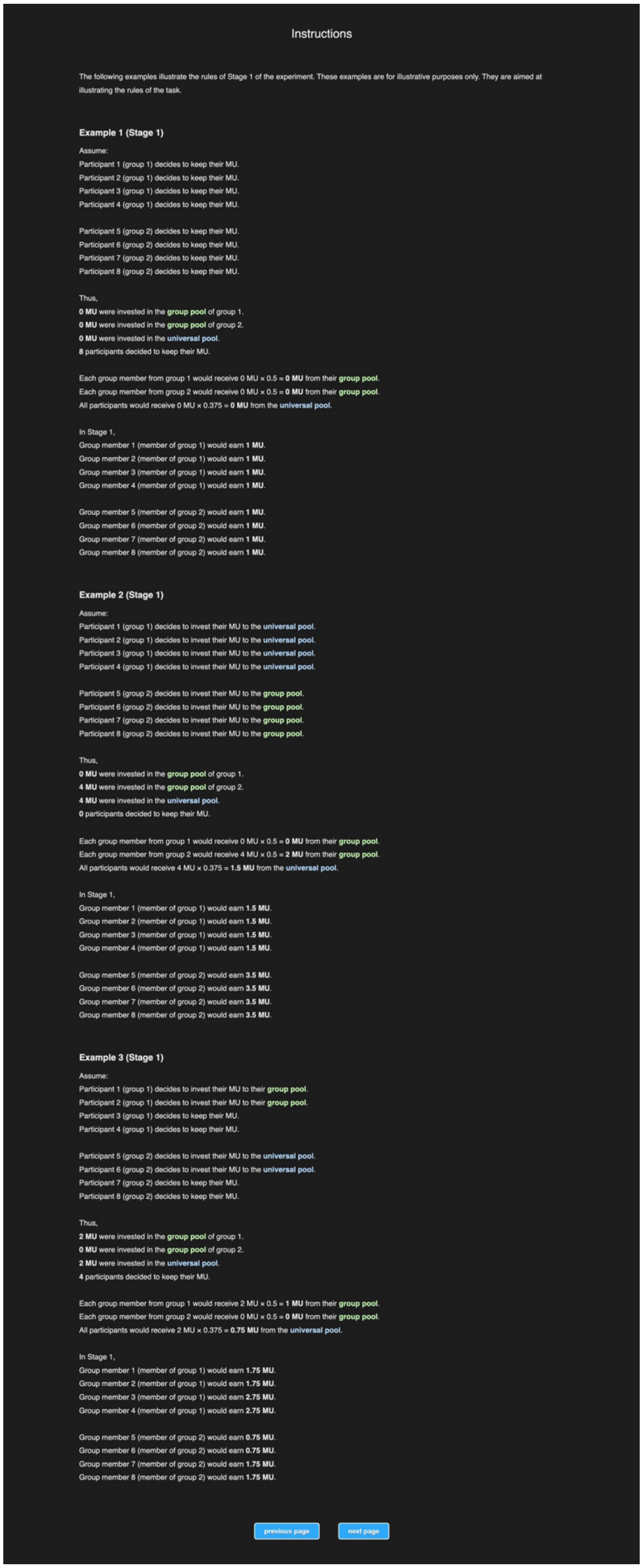

Figure S11. Examples for stage 1 (page 6).

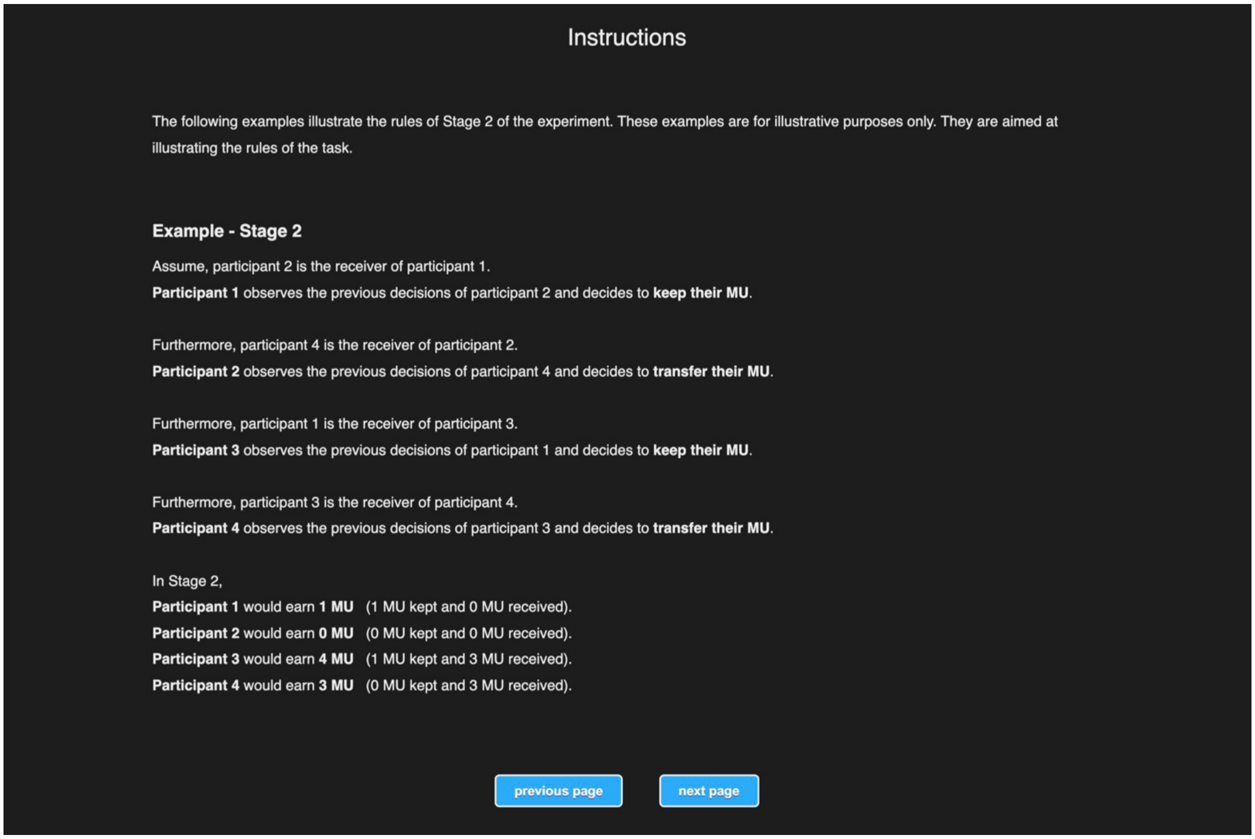

Figure S12. Examples for stage 2 (page 7).

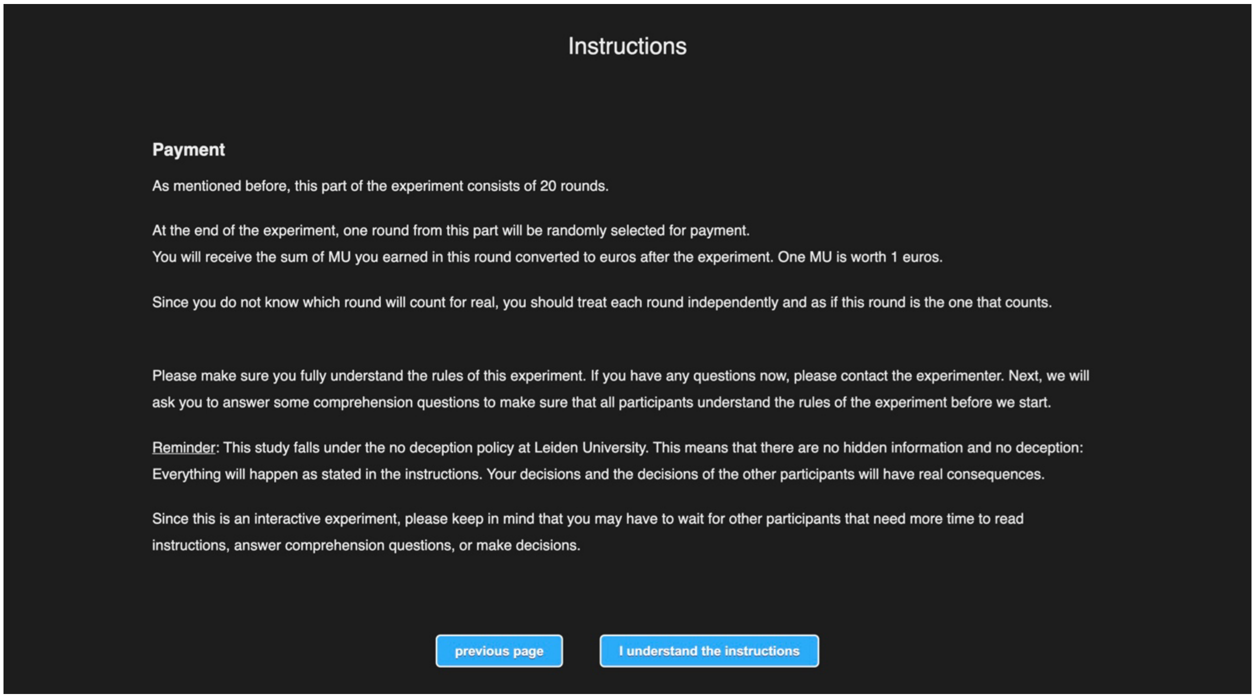

Figure S13. Instructions (page 8).

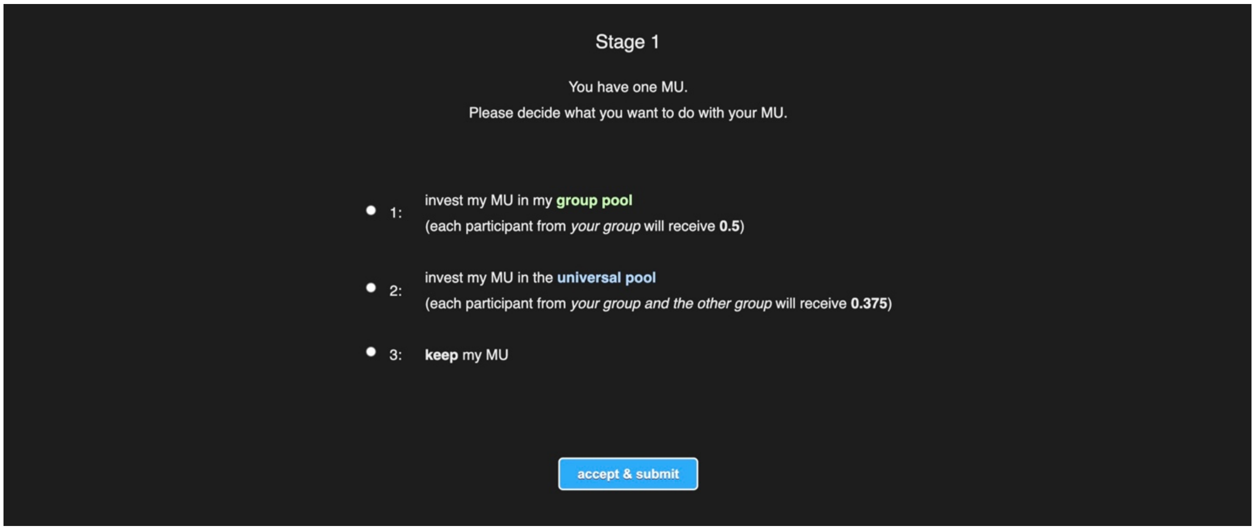

Figure S14. Interface – stage 1 decision.

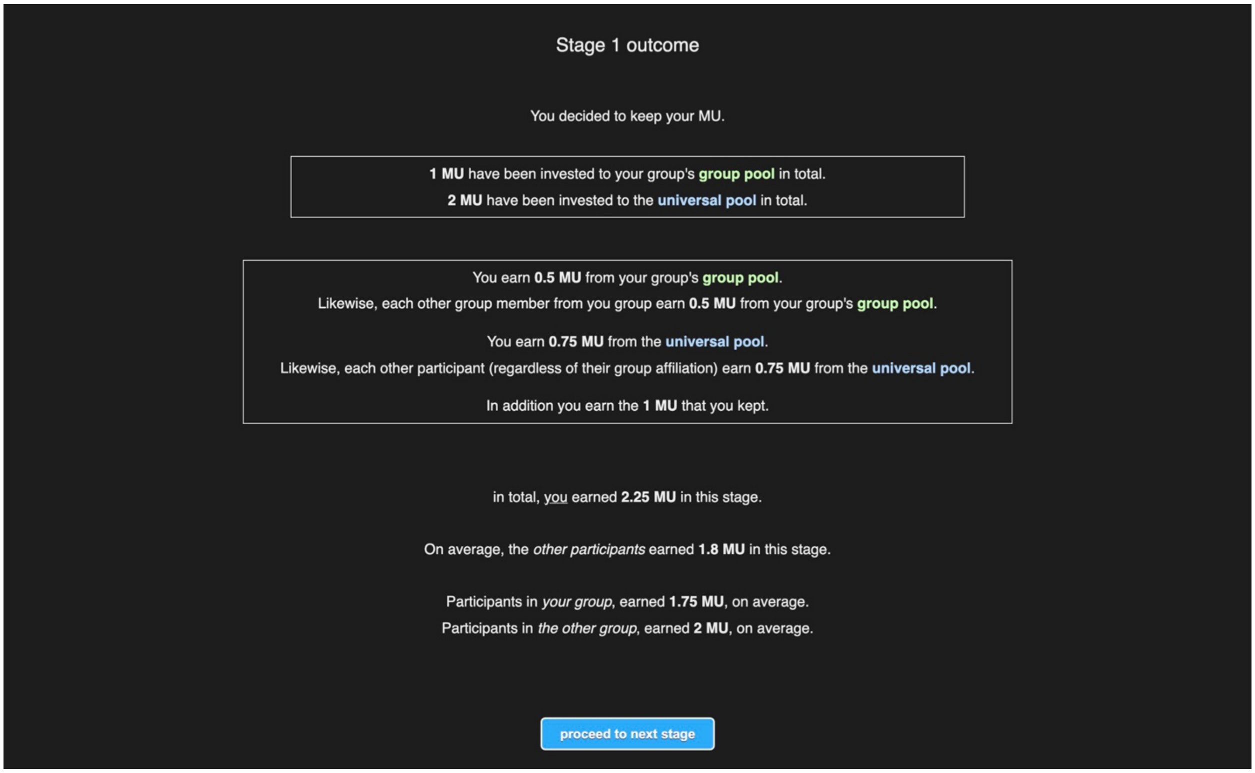

Figure S15. Interface – stage 1 exemplary feedback.

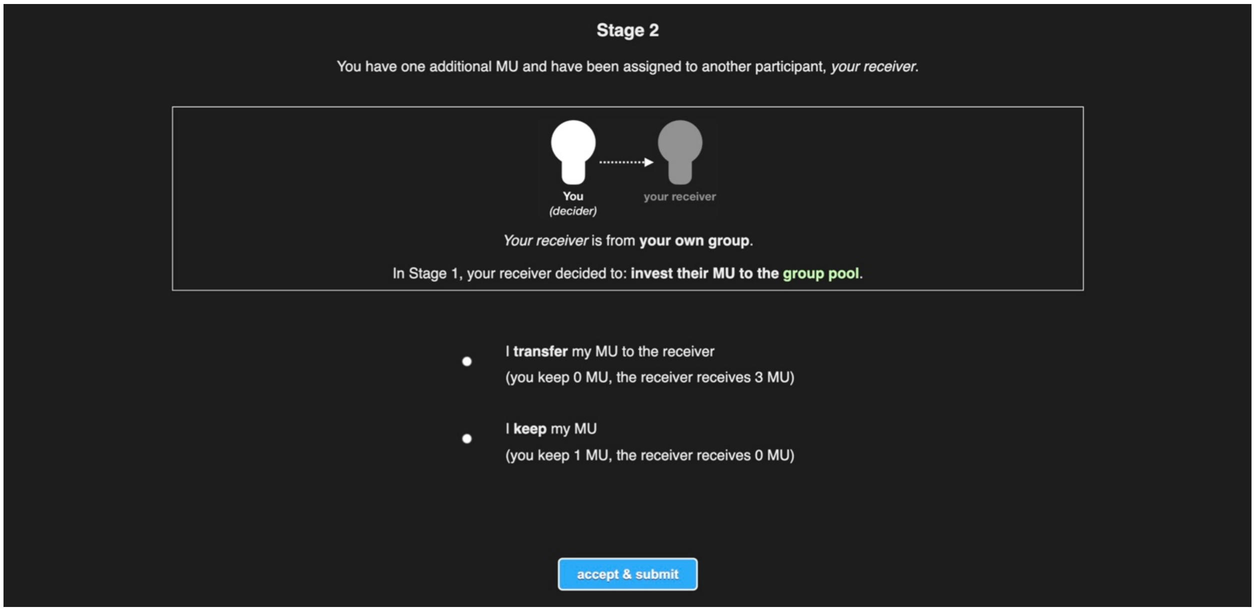

Figure S16. Interface – stage 2 decision.

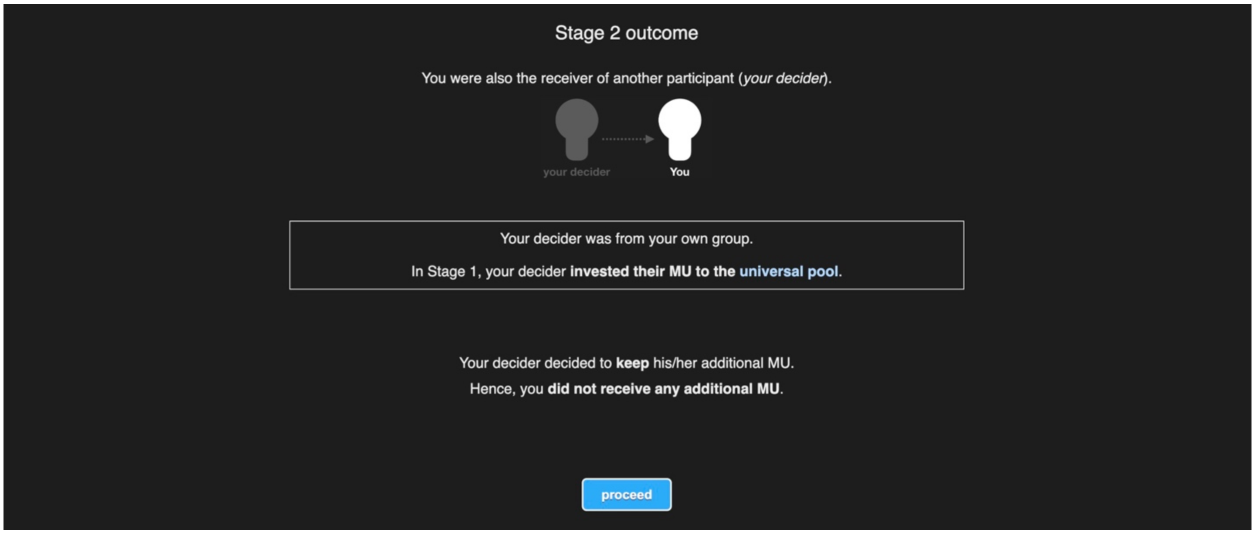

Figure S17. Interface – stage 2 exemplary feedback.

REFERENCES AND NOTES

1. J. Gross, C. K. W. De Dreu, Individual solutions to shared problems create a modern tragedy of the commons. *Sci. Adv.* **5**, eaau7296 (2019).

2. E. Fehr, I. Schurtenberger, Normative foundations of human cooperation. *Nat. Hum. Behav.* **2**, 458–468 (2018).

3. H. Aaldering, R. Böhm, Parochial versus universal cooperation: Introducing a novel economic game of within- and between-group interaction. *Soc. Psychol. Personal. Sci.* **11**, 36–45 (2020).

4. A. P. Wit, N. L. Kerr, "Me versus just us versus us all" categorization and cooperation in nested social dilemmas. *J. Pers. Soc. Psychol.* **83**, 616–637 (2002).

5. N. R. Buchan, G. Grimalda, R. Wilson, M. Brewer, E. Fatas, M. Foddy, Globalization and human cooperation. *Proc. Natl. Acad. Sci. U.S.A.* **106**, 4138–4142 (2009).

6. C. K. W. De Dreu, J. Gross, A. Fariña, Y. Ma, Group cooperation, carrying-capacity stress, and intergroup conflict. *Trends Cogn. Sci.* **24**, 760–776 (2020).

7. S. Chakravarty, M. A. Fonseca, Discrimination via exclusion: An experiment on group identity and club goods. *J. Public Econ. Theory.* **19**, 244–263 (2017).

8. J. Wang, B. Wu, D. W. C. Ho, L. Wang, Evolution of cooperation in multilevel public goods games with community structures. *Europhys. Lett.* **93**, 58001 (2011).

9. C. Gallier, T. Goeschl, M. Kesternich, J. Lohse, C. Reif, D. Römer, Leveling up? An inter-neighborhood experiment on parochialism and the efficiency of multi-level public goods provision. *J. Econ. Behav. Organ.* **164**, 500–517 (2019).

10. C. Blackwell, M. McKee, Only for my own neighborhood? Preferences and voluntary provision of local and global public goods. *J. Econ. Behav. Organ.* **52**, 115–131 (2003).

11. G. Fellner, G. K. Lünser, Cooperation in local and global groups. *J. Econ. Behav. Organ.* **108**, 364–373 (2014).

12. D. G. Victor, Toward effective international cooperation on climate change: Numbers, interests and institutions. *Glob. Environ. Polit.* **6**, 90–103 (2006).

13. T. Distefano, S. D'Alessandro, A new two-nested-game approach: Linking micro- and macro-scales in international environmental agreements. *Int. Environ. Agreem. Politics Law Econ.* **21**, 493–516 (2021).

14. E. Ostrom, J. Burger, C. B. Field, R. B. Norgaard, D. Policansky, Revisiting the Commons: Local lessons, global challenges. *Science* **284**, 278–282 (1999).

15. T. Dietz, E. Ostrom, P. C. Stern, The struggle to govern the commons. *Science* **302**, 1907–1912 (2003).

16. P. A. M. Van Lange, J. Joireman, C. D. Parks, E. Van Dijk, The psychology of social dilemmas: A review. *Organ. Behav. Hum. Decis. Process.* **120**, 125–141 (2013).

17. U. Fischbacher, S. Gächter, Social preferences, beliefs, and the dynamics of free riding in public goods experiments. *Am. Econ. Rev.* **100**, 541–556 (2010).

18. U. Fischbacher, S. Gächter, E. Fehr, Are people conditionally cooperative? Evidence from a public goods experiment. *Econ. Lett.* **71**, 397–404 (2001).

19. A. Chaudhuri, Sustaining cooperation in laboratory public goods experiments: A selective survey of the literature. *Exp. Econ.* **14**, 47–83 (2011).

20. M. A. Nowak, Five rules for the evolution of cooperation. *Science* **314**, 1560–1563 (2006).

21. D. G. Rand, M. A. Nowak, Human cooperation. *Trends Cogn. Sci.* **17**, 413–425 (2013).

22. E. Gallo, C. Yan, The effects of reputational and social knowledge on cooperation. *Proc. Natl. Acad. Sci. U.S.A.* **112**, 3647–3652 (2015).

23. S. Suzuki, E. Akiyama, Reputation and the evolution of cooperation in sizable groups. *Proc. Biol. Sci.* **272**, 1373–1377 (2005).

24. E. Ostrom, J. Walker, R. Gardner, Covenants with and without a sword: Self-governance is possible. *Am. Polit. Sci. Rev.* **86**, 404–417 (1992).

25. M. Sutter, S. Haigner, M. Kocher, Choosing the carrot or the stick? Endogenous institutional choice in social dilemma situations. *Rev. Econ. Stud.* **77**, 1540–1566 (2010).

26. A. J. Stewart, J. B. Plotkin, Small groups and long memories promote cooperation. *Sci. Rep.* **6**, 26889 (2016).

27. M. A. Nowak, K. Sigmund, Evolution of indirect reciprocity. *Nature* **437**, 1291–1298 (2005).

28. M. Milinski, D. Semmann, H.-J. Krambeck, Reputation helps solve the 'tragedy of the commons'. *Nature* **415**, 424–426 (2002).

29. M. Milinski, D. Semmann, T. C. M. Bakker, H.-J. Krambeck, Cooperation through indirect reciprocity: image scoring or standing strategy? *Proc. R. Soc. Lond. Ser. B Biol. Sci.* **268**, 2495–2501 (2001).

30. D. G. Rand, A. Dreber, T. Ellingsen, D. Fudenberg, M. A. Nowak, Positive interactions promote public cooperation. *Science* **325**, 1272–1275 (2009).

31. F. L. Pinheiro, V. V. Vasconcelos, F. C. Santos, J. M. Pacheco, Evolution of all-or-none strategies in repeated public goods dilemmas. *PLOS Comput. Biol.* **10**, e1003945 (2014).

32. H. Bernhard, U. Fischbacher, E. Fehr, Parochial altruism in humans. *Nature* **442**, 912–915 (2006).

33. A. Romano, M. Sutter, J. H. Liu, T. Yamagishi, D. Balliet, National parochialism is ubiquitous across 42 nations around the world. *Nat. Commun.* **12**, 4456 (2021).

34. J. García, J. C. J. M. van den Bergh, Evolution of parochial altruism by multilevel selection. *Evol. Hum. Behav.* **32**, 277–287 (2011).

35. H. Bernhard, E. Fehr, U. Fischbacher, Group affiliation and altruistic norm enforcement. *Am. Econ. Rev.* **96**, 217–221 (2006).

36. F. Fu, C. E. Tarnita, N. A. Christakis, L. Wang, D. G. Rand, M. A. Nowak, Evolution of in-group favoritism. *Sci. Rep.* **2**, 460 (2012).

37. R. M. Whitaker, G. B. Colombo, D. G. Rand, Indirect reciprocity and the evolution of prejudicial groups. *Sci. Rep.* **8**, 13247 (2018).

38. R. Thomson, M. Yuki, T. Talhelm, J. Schug, M. Kito, A. H. Ayanian, J. C. Becker, M. Becker, C. Chiu, H.-S. Choi, C. M. Ferreira, M. Fülöp, P. Gul, A. M. Houghton-Illera, M. Joasoo, J. Jong, C. M. Kavanagh, D. Khutkyy, C. Manzi, U. M. Marcinkowska, T. L. Milfont, F. Neto, T. von Oertzen, R. Pliskin, A. S. Martin, P. Singh, M. L. Visserman, Relational mobility predicts social behaviors in 39 countries and is tied to historical farming and threat. *Proc. Natl. Acad. Sci. U.S.A.* **115**, 7521–7526 (2018).

39. M. Yuki, J. Schug, Psychological consequences of relational mobility. *Curr. Opin. Psychol.* **32**, 129–132 (2020).

40. V. Grimm, F. Mengel, Cooperation in viscous populations—Experimental evidence. *Games Econ. Behav.* **66**, 202–220 (2009).

41. M. Dyble, G. D. Salali, N. Chaudhary, A. Page, D. Smith, J. Thompson, L. Vinicius, R. Mace, A. B. Migliano, Sex equality can explain the unique social structure of hunter-gatherer bands. *Science* **348**, 796–798 (2015).

42. H. M. Lewis, L. Vinicius, J. Strods, R. Mace, A. B. Migliano, High mobility explains demand sharing and enforced cooperation in egalitarian hunter-gatherers. *Nat. Commun.* **5**, 5789 (2014).

43. J. Scholte, *Globalization: A Critical Introduction* (Bloomsbury Publishing, 2017).

44. M. A. Nowak, K. Sigmund, Evolution of indirect reciprocity by image scoring. *Nature* **393**, 573–577 (1998).

45. H. H. Nax, M. Perc, A. Szolnoki, D. Helbing, Stability of cooperation under image scoring in group interactions. *Sci. Rep.* **5**, 12145 (2015).

46. K. Panchanathan, R. Boyd, Indirect reciprocity can stabilize cooperation without the second-order free rider problem. *Nature* **432**, 499–502 (2004).

47. I. Okada, A review of theoretical studies on indirect reciprocity. *Games* **11**, 27 (2020).

48. B. Rockenbach, M. Milinski, The efficient interaction of indirect reciprocity and costly punishment. *Nature* **444**, 718–723 (2006).

49. O. P. Hauser, A. Hendriks, D. G. Rand, M. A. Nowak, Think global, act local: Preserving the global commons. *Sci. Rep.* **6**, 36079 (2016).

50. D. G. Rand, M. A. Nowak, The evolution of antisocial punishment in optional public goods games. *Nat. Commun.* **2**, 434 (2011).

51. J. García, A. Traulsen, Evolution of coordinated punishment to enforce cooperation from an unbiased strategy space. *J. R. Soc. Interface* **16**, 20190127 (2019).

52. J. Gross, C. K. W. De Dreu, The rise and fall of cooperation through reputation and group polarization. *Nat. Commun.* **10**, 776 (2019).

53. N. R. Buchan, M. B. Brewer, G. Grimalda, R. K. Wilson, E. Fatas, M. Foddy, Global social identity and global cooperation. *Psychol. Sci.* **22**, 821–828 (2011).

54. S. L. Gaertner, J. A. Mann, J. F. Dovidio, A. J. Murrell, M. Pomare, How does cooperation reduce intergroup bias? *J. Pers. Soc. Psychol.* **59**, 692–704 (1990).

55. J. Gross, S. Veistola, C. K. W. De Dreu, E. Van Dijk, Self-reliance crowds out group cooperation and increases wealth inequality. *Nat. Commun.* **11**, 5161 (2020).

56. J. Gross, R. Böhm, Voluntary restrictions on self-reliance increase cooperation and mitigate wealth inequality. *Proc. Natl. Acad. Sci. U.S.A.* **117**, 29202–29211 (2020).

57. J. Berg, J. Dickhaut, K. McCabe, Trust, reciprocity, and social history. *Games Econ. Behav.* **10**, 122–142 (1995).

58. R. O. Murphy, K. A. Ackermann, M. Handgraaf, Measuring social value orientation. *Judgm. Decis. Mak.* **6**, 771–781 (2011).

59. C. W. Leach, M. van Zomeren, S. Zebel, M. L. W. Vliek, S. F. Pennekamp, B. Doosje, J. W. Ouwerkerk, R. Spears, Group-level self-definition and self-investment: A hierarchical (multicomponent) model of in-group identification. *J. Pers. Soc. Psychol.* **95**, 144–165 (2008).

60. J. L. Pletzer, D. Balliet, J. Joireman, D. M. Kuhlman, S. C. Voelpel, P. A. M. Van Lange, Social value orientation, expectations, and cooperation in social dilemmas: A meta-analysis. *Eur. J. Personal.* **32**, 62–83 (2017).
